# Cancer-associated fibroblasts require proline synthesis by PYCR1 for the deposition of pro-tumorigenic extracellular matrix

**DOI:** 10.1038/s42255-022-00582-0

**Published:** 2022-06-27

**Authors:** Emily J. Kay, Karla Paterson, Carla Riera-Domingo, David Sumpton, J. Henry M. Däbritz, Saverio Tardito, Claudia Boldrini, Juan R. Hernandez-Fernaud, Dimitris Athineos, Sandeep Dhayade, Ekaterina Stepanova, Enio Gjerga, Lisa J. Neilson, Sergio Lilla, Ann Hedley, Grigorios Koulouras, Grace McGregor, Craig Jamieson, Radia Marie Johnson, Morag Park, Kristina Kirschner, Crispin Miller, Jurre J. Kamphorst, Fabricio Loayza-Puch, Julio Saez-Rodriguez, Massimiliano Mazzone, Karen Blyth, Michele Zagnoni, Sara Zanivan

**Affiliations:** 1 Cancer Research UK Beatson Institute, Glasgow, UK; 2 Institute of Cancer Sciences, University of Glasgow, Glasgow, UK; 3 Centre for Microsystems and Photonics, EEE Department, University of Strathclyde, Glasgow, UK; 4 Laboratory of Tumor Inflammation and Angiogenesis, Center for Cancer Biology (CCB), VIB, Leuven, Belgium; 5 Laboratory of Tumor Inflammation and Angiogenesis, Department of Oncology, KU Leuven, Leuven, Belgium; 6 Translational Control and Metabolism, German Cancer Research Center (DKFZ), Heidelberg, Germany; 7 Heidelberg University, Faculty of Medicine, Institute for Computational Biomedicine, Bioquant, Heidelberg, Germany; 8 RWTH Aachen University, Faculty of Medicine, Joint Research Centre for Computational Biomedicine (JRC-COMBINE), Aachen, Germany; 9 Department of Pure and Applied Chemistry, Thomas Graham Building, University of Strathclyde, Glasgow, UK; 10 Rosalind and Morris Goodman Cancer Research Centre, McGill University, Montreal, Quebec Canada; 11 Department of Biochemistry, McGill University, Montreal, Quebec Canada; 12 Department of Medicine, McGill University, Montreal, Quebec Canada; 13 Department of Oncology, McGill University, Montreal, Quebec Canada

**Keywords:** Cancer microenvironment, Cancer metabolism, Metabolism

## Abstract

Elevated production of collagen-rich extracellular matrix is a hallmark of cancer-associated fibroblasts (CAFs) and a central driver of cancer aggressiveness. Here we find that proline, a highly abundant amino acid in collagen proteins, is newly synthesized from glutamine in CAFs to make tumour collagen in breast cancer xenografts. PYCR1 is a key enzyme for proline synthesis and highly expressed in the stroma of breast cancer patients and in CAFs. Reducing PYCR1 levels in CAFs is sufficient to reduce tumour collagen production, tumour growth and metastatic spread in vivo and cancer cell proliferation in vitro. Both collagen and glutamine-derived proline synthesis in CAFs are epigenetically upregulated by increased pyruvate dehydrogenase-derived acetyl-CoA levels. PYCR1 is a cancer cell vulnerability and potential target for therapy; therefore, our work provides evidence that targeting PYCR1 may have the additional benefit of halting the production of a pro-tumorigenic extracellular matrix. Our work unveils new roles for CAF metabolism to support pro-tumorigenic collagen production.

## Abstract (web summary)

Targeting extracellular matrix (ECM) remodelling is a feasible avenue to prevent cancer aggressiveness and metastasis. In this study, Kay et al. show that metabolic flux in cancer-associated fibroblasts is coupled to enhanced proline synthesis by PYCR1 to support elevated production of collagen-rich ECM, thus contributing to cancer spreading.

## Main

CAFs are mesenchymal cells abundant in the stroma of solid tumours and active players in tumour initiation, progression, metastasis and response to anti-cancer therapies^1,2^. Because of the extensive body of evidence that CAFs support cancer, targeting CAFs has emerged as a unique opportunity to control cancer.

Distinct functional subpopulations of CAFs coexist in the stroma of solid tumours^1^ . Among those, myofibroblast-like CAFs (myCAFs) produce an abundant collagen-rich extracellular matrix (ECM) and express high levels of alpha smooth muscle actin (ACTA2 or αSMA)^1^. The presence of myCAF in the tumour stroma has been described in human and murine solid cancers^1,3^. In patients, αSMA-expressing CAF abundance is an indicator of poor prognosis in several cancer types^4,5^, including breast cancer^6,7^. Moreover, CAFs isolated from patients and kept in culture retain myCAF features^6–8^ and when cotransplanted with cancer cells accelerate tumour growth and progression^7,9^. In contrast, the depletion of proliferating αSMA-expressing CAFs^10^ or inhibition of their expansion^11^ accelerated cancer and promoted metastasis. Therefore, controlling cancer progression may not require CAFs to be killed, but rather the targeting of specific molecules and pathways that control their pro-tumorigenic functions.

Targeting ECM production is also a potential therapeutic strategy, because the composition and mechanical properties of the ECM are established active drivers of tumour pathology^12,13^. Collagen is the most abundant component of the tumour ECM. Genetic depletion of collagen VI (*Col6a1*) reduced the rate of tumour initiation and growth, while overexpression of collagen I (*Col1a1*) in breast cancer models has been shown to enhance tumour formation and progression and increase the incidence of metastasis^14–16^. Collagen also contributes to the impediment of effective delivery of therapeutics and immune cell recruitment by hampering the growth of functional tumour vasculature^17–19^. However, reducing tumour collagen production by deleting *Col1a1* in αSMA-expressing cells or upon treatment with anti–lysyl oxidase like-2 antibody accelerated progression of pancreatic cancer^20,21^, suggesting that a collagen-rich ECM may also have tumour-protecting functions. This ambiguity underlines the need for a better understanding of the molecular mechanisms that underpin the production of pro-tumorigenic ECM to develop complementary and different approaches that target ECM production in cancer.

Metabolic reprogramming is another feature of CAF activation that has been shown to have tumour-promoting functions, primarily by providing tumour cells with nutrients^22–25^. However, how to effectively target CAF metabolism in tumours is still an open question, since metabolic vulnerabilities of CAFs and cancer cells can be different, and crosstalk between the cell types creates an intertwined metabolic network. A deeper understanding of CAF metabolic reprogramming is needed to identify vulnerabilities with which to target CAF activation and tumour progression.

Using a previously characterized model of tumour-promoting mammary CAFs^8,9^ and patient-derived CAFs isolated from aggressive breast tumours and their matched normal fibroblasts (NFs) isolated from tumour-adjacent tissue, we show that the production of pro-tumorigenic collagens requires increased proline synthesis from glutamine. Moreover, we show how a key enzyme for proline synthesis, pyrroline-5-carboxylate reductase 1 (PYCR1), acetyl-CoA levels and the epigenetic regulator histone acetyl-transferase EP300 are major regulators of this process.

## Results

### CAFs synthesize proline for collagen production in vitro and in vivo

**Fig. 1:**
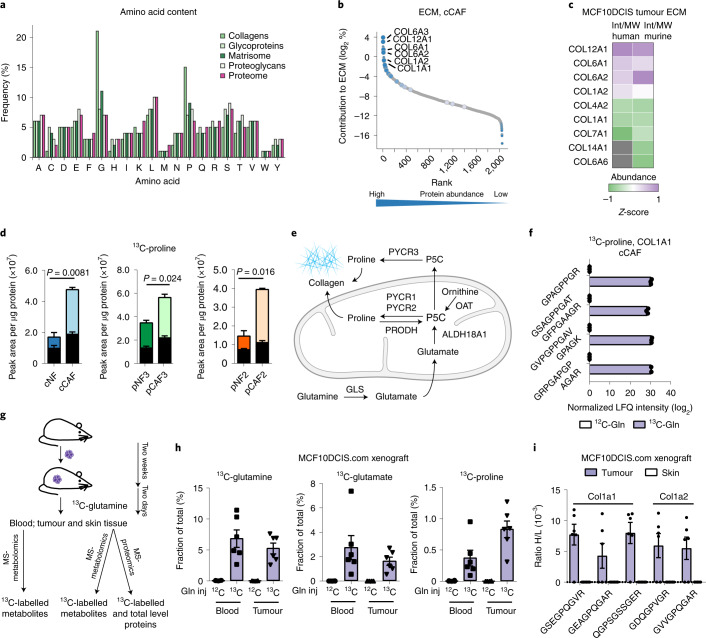
CAFs use glutamine-derived proline for collagen synthesis. **a**, Average frequency of occurrence of each amino acid in proteins in the human proteome (purple), matrisome (dark green) and matrisome components (other greens) as defined by Naba et al.^26^. **b**, Estimated total abundance (intensity/molecular weight used to rank proteins from low to high abundant, *x* axis) of proteins identified in the cCAF ECM as measured by MS-proteomics^8^. The contribution of each protein to the total mass of the ECM is shown on the *y* axis. Highlighted are the most abundant protein collagens. **c**, Comparison of the abundance (intensity/molecular weight (MW)) of collagen proteins, measured by MS-proteomics, between endogenous (murine) and transplanted (human) stroma in xenograft tumours of MCF10DCIS.com cells cotransplanted with pCAF2 fibroblasts and grown for two weeks in immunocompromised Balb/c mice. *Z*-scoring of the protein intensities was performed separately for murine and human collagens. *n* = 12 mice. **d**, Total ^13^C-labelled (coloured bar) and unlabelled (black bar) proline in mammary NFs and CAFs labelled with ^13^C-glutamine, measured by MS-metabolomics. *n* = 3 biological replicates. **e**, Scheme showing proline biosynthesis pathways from glutamine and ornithine. **f**, MS-proteomic analysis of ECM derived from cCAFs labelled for 72 h with 2 mM ^13^C-glutamine (^13^C-gln) or ^12^C-glutamine (^12^C-gln), showing ^13^C-proline incorporation into COL1A1 peptides. *n* = 3 biological replicates. **g**, Workflow of the MCFDCIS.com xenografts with ^13^C_5_-glutamine tracing and MS-based tissue analyses. **h**, Proportion of ^13^C-labelled glutamine, glutamate and proline in the blood and tumours of mice with MCF10DCIS.com xenografts after 48 h of treatment with ^13^C-glutamine or ^12^C-glutamine. 0 means that no heavy labelled amino acid had been detected at the MS. *n* = 6 mice for each treatment. Gln inj, Glutamine injection. **i**, MS-proteomic analysis of tumours and skin from **h** showing ^13^C-proline incorporation into murine Col1a1 and Col1a2 peptides. H/L, ratio Heavy (13C)/Light (12C). Error bars indicate mean ± s.e.m. Error bars indicate mean ± s.e.m. *P* values were calculated with two-tailed unpaired *t*-test with Welch’s correction. Source data

The production of abundant collagen-rich ECM is a trait that CAFs acquire during transition from normal to activated fibroblasts^2^. Amino acid frequency analysis of the matrisome^26^ and total cell proteome pinpointed an exceptionally high content of proline and glycine residues in collagen proteins (Fig. 1a and Supplementary Data 1). This is in line with the common knowledge that collagen proteins contain repeating glycine–proline–hydroxyproline sequences that allow them to fold and assemble into stable fibres^27^, and raises the question of how CAFs can metabolically support this increased biosynthetic demand.

**Extended Data Fig. 1:**
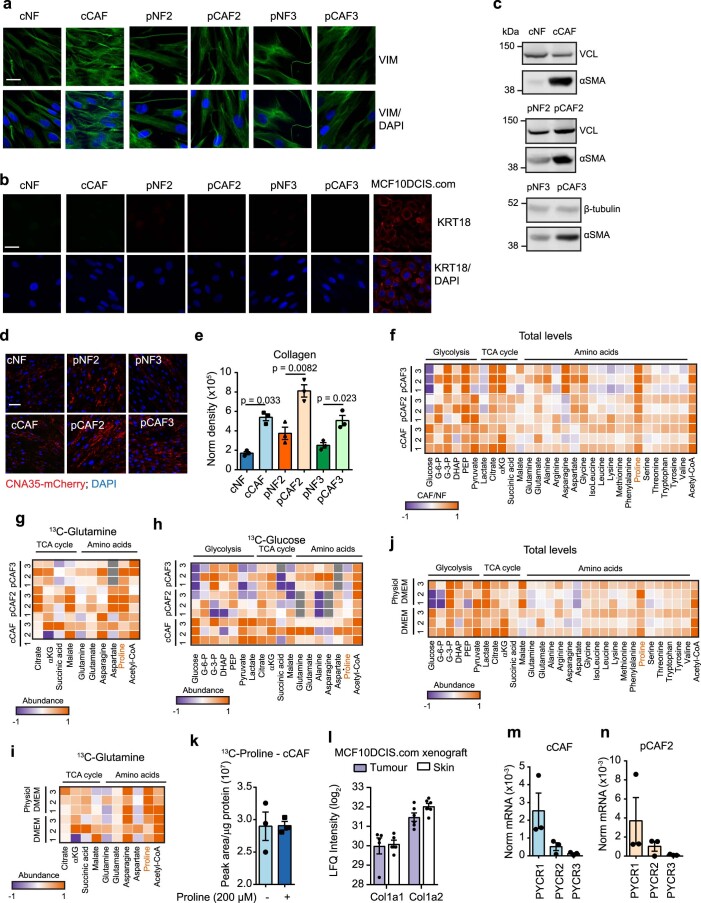
Characterisation of mammary CAF/NF pairs. **a**. Representative of 2 confocal microscopy images of vimentin staining in mammary CAF and NF pairs. Scale bar = 20 µm. **b**. Representative of 3 confocal microscopy images of keratin 18 (KRT18) staining in mammary CAF and NF pairs and MCF10DCIS.com breast cancer cells. Scale bar = 20 µm. **c**. Representative of >3 western blots showing α-SMA levels in mammary NFs and CAFs. **d, e**. Representative images (d) and quantification (e) of collagen produced by mammary NFs and CAFs. p-values were calculated with a 2-tailed unpaired t-test with Welch’s correction. Scale bar = 50 µm. N = 3 biological replicates. **f**. Fold change in total levels of glycolysis, TCA cycle and amino acid metabolites measured by MS analysis between paired CAFs and NFs. N = 3 biological replicates. **g**. Fold change in ^13^C-Glutamine labelled glycolysis, TCA cycle and amino acid metabolites measured by MS analysis between paired CAFs and NFs. N = 3 biological replicates. **h**. Fold change in ^13^C-Glucose labelled glycolysis, TCA cycle and amino acid metabolites measured by MS analysis between paired CAFs and NFs. N = 3 biological replicates. **i**. Fold change in ^13^C-Glutamine labelled metabolites between cCAFs cultured in standard DMEM with ^13^C-Glutamine or physiological DMEM (5 mM glucose, 0.65 mM glutamine, 100 µM pyruvate, 100 µM acetate) with ^13^C-Glutamine measured by MS-metabolomics. N = 3 biological replicates. **j**. Fold change in total levels of metabolites between cCAFs cultured in standard DMEM or physiological DMEM, measured by MS-metabolomics. N = 3 biological replicates. **k**. Total ^13^C-labelled proline in cCAFs cultured in physiological DMEM with ^13^C-Glutamine ± 200 µM proline, measured by MS-metabolomics. N = 3 biological replicates. **l**. Murine Col1a1 and Col1a2 protein levels in the tumour and skin tissues of mice carrying MCF10DCIS.com xenografts. N = 6 mice. **m**. mRNA levels of PYCR1, PYCR2 and PYCR3 in cCAFs, normalised to 18S levels. N = 3 biological replicates. **n**. mRNA levels of PYCR1, PYCR2 and PYCR3 in pCAF2 measured by qPCR and normalised to 18S levels. N = 3 biological replicates. Error bars indicate mean ± SEM. Source data

To address this question, we used a previously established model of tumour-promoting mammary CAFs and their NF parental line^8,9^ (referred to as cCAF and cNF, respectively), and two lines of patient-derived CAFs (pCAFs) and their matched normal fibroblasts (pNFs). cCAFs were derived from immortalized human normal mammary fibroblasts that were activated when cotransplanted with human breast cancer cells in subcutaneous tumours^9^, while we generated pCAFs and pNFs from surgical samples of patients with breast cancer at an advanced stage. All the fibroblasts expressed the mesenchymal marker vimentin but not the epithelial marker keratin 18 (Extended Data Fig. 1a,b). Moreover, both cCAFs and pCAFs have a myofibroblast-like phenotype, producing high levels of collagen and αSMA compared to matched NFs^8,9^ (Extended Data Fig. 1c–e). Our previous proteomic analysis of ECM derived from cCAFs in culture^8^ showed that collagen contributes over 30% of the total ECM, and that COL12A, COL6A and COL1A are the most abundant collagen proteins (Fig. 1b and Supplementary Data 2). To assess whether CAFs produce similar ECM in vivo, we performed a mass spectrometry (MS)-proteomic analysis of xenografts grown in Balb/c nude mice following subcutaneous cotransplantation of pCAF and MCF10DCIS.com breast cancer cells, a model in which CAFs accelerate tumour growth^28^. COL12A, COL6A and COL1A were the most abundant collagens produced by both endogenous (murine) and transplanted (human) tumour stroma (Fig. 1c and Supplementary Data 3, datasheet ‘Intensity collagens tum’). As a result, our CAF lines produce abundant amounts of collagen I and VI, both of which have been shown to promote breast cancer progression and are relevant models to study the role of CAF-derived collagen in tumours.

MS-metabolomics analysis of intracellular metabolites in mammary CAFs and NFs pinpointed proline as being consistently more abundant in CAFs (Extended Data Fig. 1f). MS-based tracing experiments of cells labelled for 24 hours with ^13^C_6_-glucose or ^13^C_5_-glutamine showed that CAFs synthesized more proline from glutamine (Fig. 1d and Extended Data Fig. 1g,h). Using the cCAF line, we also found that culturing them in Dulbecco’s modified Eagle’s medium (DMEM) containing physiological levels of glucose (5 mM) and glutamine (0.65 mM) (physiol. DMEM) had a minimal impact on the total levels of intracellular proline and did not reduce proline synthesis (Extended Data Fig. 1i,j). Furthermore, proline synthesis from glutamine was not altered when physiological levels of proline (200 µM^29^) were added to the medium (Extended Data Fig. 1k). It should be noted that we cultured fibroblasts in physiological levels of glycine and supraphysiological levels of serine, so it is possible that when glycine or serine are limited CAFs also increase glycine synthesis, which is highly abundant in collagen. Therefore, CAFs have an enhanced ability to synthesize proline, one of the most abundant amino acids in collagen proteins.

To determine whether CAFs utilize glutamine-derived proline to make collagen (Fig. 1e) we cultured them in medium with ^13^C_5_-glutamine and monitored the presence of ^13^C_5_-proline in collagen proteins by MS-proteomic analysis of their ECM. MS analysis detected ^13^C_5_-proline in COL1A1 peptides (Fig. 1f and Supplementary Data 4), demonstrating that CAFs use newly synthesized proline to make collagen. To explore whether this was also true in vivo, we traced ^13^C_5_-glutamine in MCF10DCIS.com tumour-bearing Balb/c nude mice (Fig. 1g). ^13^C_5_-glutamine, ^13^C_5_-glutamate and ^13^C_5_-proline were detected in blood and tumour tissue (Fig. 1h). While the enrichment of ^13^C-glutamine and ^13^C-glutamate was comparable in the blood and the tumour, ^13^C_5_-proline was more abundant in the tumour than in the blood, demonstrating that the in situ synthesis of proline contributes to the intratumoural proline pool. Furthermore, MS-proteomic analysis of the tumours detected Col1a1 and Col1a2 peptides containing ^13^C_5_-proline (Fig. 1i and Supplementary Data 3, datasheet ‘H/L ratio collagen peptides’). Together these data provide evidence that circulating glutamine is used to supply proline for tumour collagen synthesis in vivo. Interestingly, we could not detect ^13^C_5_-proline-containing Col1a peptides in the skin (Fig. 1i), even though the total levels of collagen were similar to those in the tumours (Extended Data Fig. 1l, Supplementary Data 3, datasheets ‘LFQ intensity collagens tum-skin’ and ‘H/L ratio collagen peptides’). This is in agreement with the low rate of proline synthesis from glutamine observed in most tissues in non-tumour-bearing mice^30^ or suggests a higher rate of collagen synthesis in tumours than in skin. Hence, increased proline synthesis from glutamine is an acquired trait of CAFs and may support collagen production in tumours.

### PYCR1 is upregulated in CAFs and breast cancer stroma

**Fig. 2:**
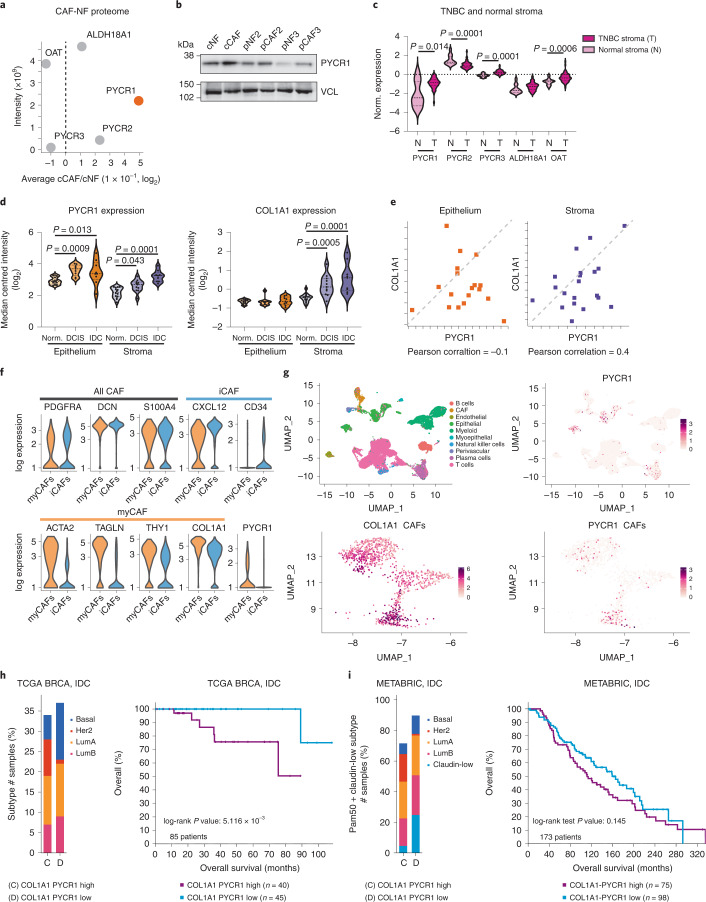
Collagen producing CAFs and stroma express high levels of PYCR1. **a**, Fold change in levels of proline synthesis enzymes between cCAFs and cNFs in an MS-proteomic analysis. The intensities of each protein, as the mean of their abundance in the fibroblasts, are shown on the *y* axis. *n* = 4 biological replicates. **b**, Representative western blot showing PYCR1 levels in paired CAFs and NFs. Vinculin (VCL) was used as a loading control. **c**, Expression of proline synthesis pathway and stromal markers in LCMD sections of norm. (normal) breast stroma and TNBC-associated stroma from Saleh et al.^31^. Significance was calculated with two-tailed unpaired *t*-tests with Welch’s correction. **d**, Violin plots showing the expression levels of *PYCR1* (probe g5902035_3p_a_at) and *COL1A1* (probe Hs.172928.0.A2_3p_a_at) in laser-captured microdissected normal breast, DCIS and IDC stroma and epithelium from the Ma et al. dataset^32^. Quantitative data downloaded from Oncomine. Median and quartiles are indicated with dashed lines. Significance was calculated with one-way ANOVA test with Sidak’s multiple comparison test. **e**, Gene expression correlation (Pearson) between PYCR1 and COL1A1 in IDC patients shown in **d**. **f**, Violin plots showing the expression of markers commonly associated to myCAF, iCAF (inflammatory CAF) and total CAF, and of PYCR1 in TNBC tumours from Wu et al.^3^. **g**, UMAP visualization of stromal, immune and cancer cells (top plots) aligned using canonical correlation analysis in Seurat. Top left, cells are coloured by their cell type annotation from Wu et al.^3^. Bottom panels contain only CAFs, as defined by Wu et al. **h**,**i**, Kaplan–Meier plots comparing overall survival of patients with IDC tumours expressing high or low levels of both *COL1A1* and *PYCR1*. On the left of each curve is shown the distribution of breast cancer subtypes in the two subsets of patients. Data generated with cBioportal using TCGA Pan Cancer Atlas (**h**) and METABRIC (**i**). Source data

**Extended Data Fig. 2:**
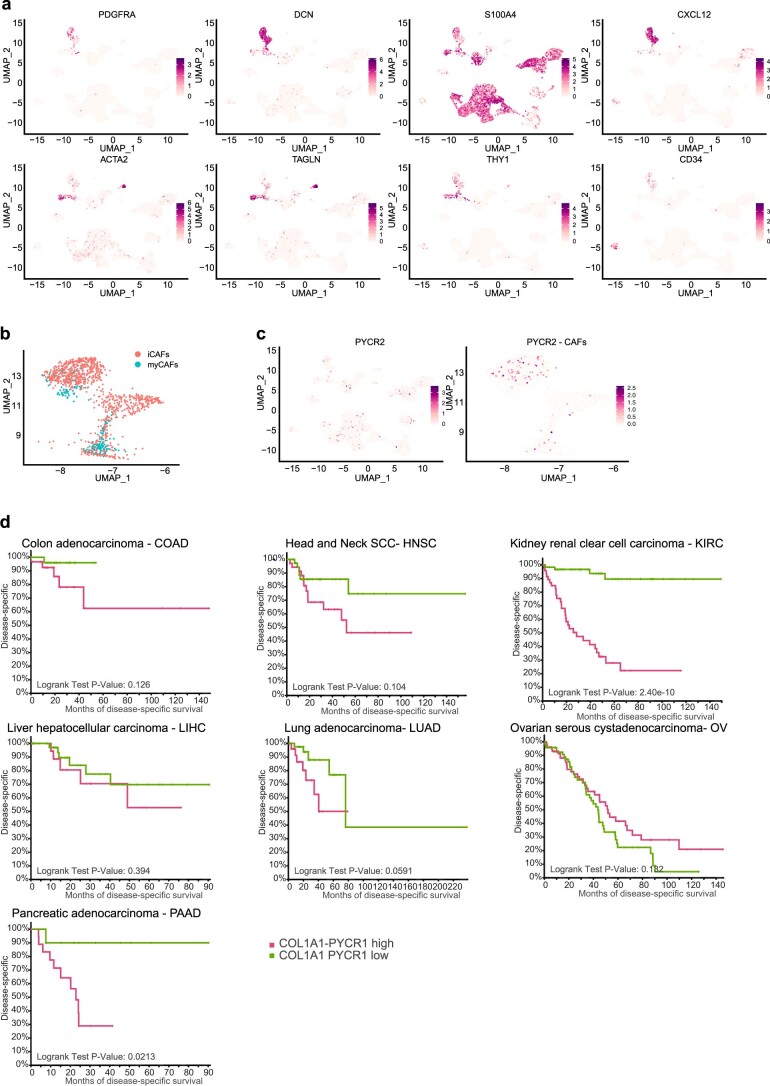
PYCR1 and COL1A1 expression in CAFs and cancer patients. **a**. UMAP visualisation of stromal, immune and cancer cells aligned using canonical correlation analysis in Seurat. Cell types defined as in Wu et al. **b**. UMAP visualisation of CAFs, with iCAF and myCAF as defined in Wu et al. **c**. UMAP visualisation of CAFs, as defined in Wu et al., aligned using canonical correlation analysis in Seurat. **d**. Kaplan-Meier plots comparing the disease specific survival of patients with tumours expressing high (top quartile) or low (bottom quartile) levels of both *COL1A1* and *PYCR1*. Data generated with cBioportal using TCGA Pan Cancer Atlas for the indicated tumour types. Source data

To identify enzymes of the proline biosynthetic pathway responsible for increased proline synthesis in CAFs (Fig. 1e), we measured the total proteome of cCAFs and cNFs (Supplementary Data 5). PYCR1–3 enzymes catalyse the last step in proline biosynthesis, by converting pyrroline-5-carboxylate into proline. PYCR1 was the most upregulated enzyme from the proline biosynthetic pathway in cCAFs (Fig. 2a). *PYCR1* messenger RNA levels were also higher compared to *PYCR2* and *PYCR3* in CAFs (Extended Data Fig. 1m,n). Western blot analysis confirmed PYCR1 upregulation in all CAF lines compared to their matched NFs (Fig. 2b). Gene expression analysis of laser capture microdissected (LCMD) stroma microdissected from normal breast and tumour from patients with advanced triple-negative breast cancers (TNBC)^31^ showed that, as in CAFs, *PYCR1* was the most upregulated proline synthesizing enzyme in tumour stroma (Fig. 2c). Moreover, available gene expression data of patient-matched microdissected stroma and epithelium from normal breast, ductal carcinoma in situ (DCIS) and invasive ductal carcinoma (IDC)^32^ showed that *PYCR1* expression was substantially higher in DCIS and further increased in IDC, both in the stroma and epithelium. *COL1A1* expression increased with disease progression, and positively correlated with *PYCR1* expression in the stroma but not the epithelium (Fig. 2d,e). To determine which cell types contribute to stromal *PYCR1* expression, we analysed a publicly available single-cell RNA sequencing (scRNA-seq) TNBC dataset^3^. Among non-epithelial cells, *PYCR1* was expressed in plasma cells and CAFs, particularly in a subset of myCAFs that express higher levels of *COL1A1* (Fig. 2f,g and Extended Data Fig. 2a,b). In contrast, *PYCR2* was expressed by few CAFs (Extended Data Fig. 2c), in line with our in vitro data (Fig. 2a).

We further explored whether high *PYCR1* and *COL1A1* expression correlated with clinical outcomes in patients with breast cancer. We analysed a cohort of 780 patients with IDC from the Pan Cancer Atlas study of The Cancer Genome Atlas (TCGA)^33^. Around 10% of patients expressed either high levels (upper quartile) of both *COL1A1* and *PYCR1*, or low levels (lower quartile) of the two genes. The subset with higher levels included a larger proportion of HER2+ tumours and patients had significantly worse outcomes (Fig. 2h). We observed similar results analysing patients with IDC from the METABRIC study^34^ (Fig. 2i), and a similar trend of worse outcomes in other tumour types expressing high *PYCR1* and *COL1A1* (Extended Data Fig. 2d).

Together, these data provide a link between PYCR1 and collagen in CAFs and breast cancer stroma. Based on these premises, we investigated the role of PYCR1 in collagen production in CAFs.

### PYCR1 provides proline for collagen biosynthesis

**Fig. 3:**
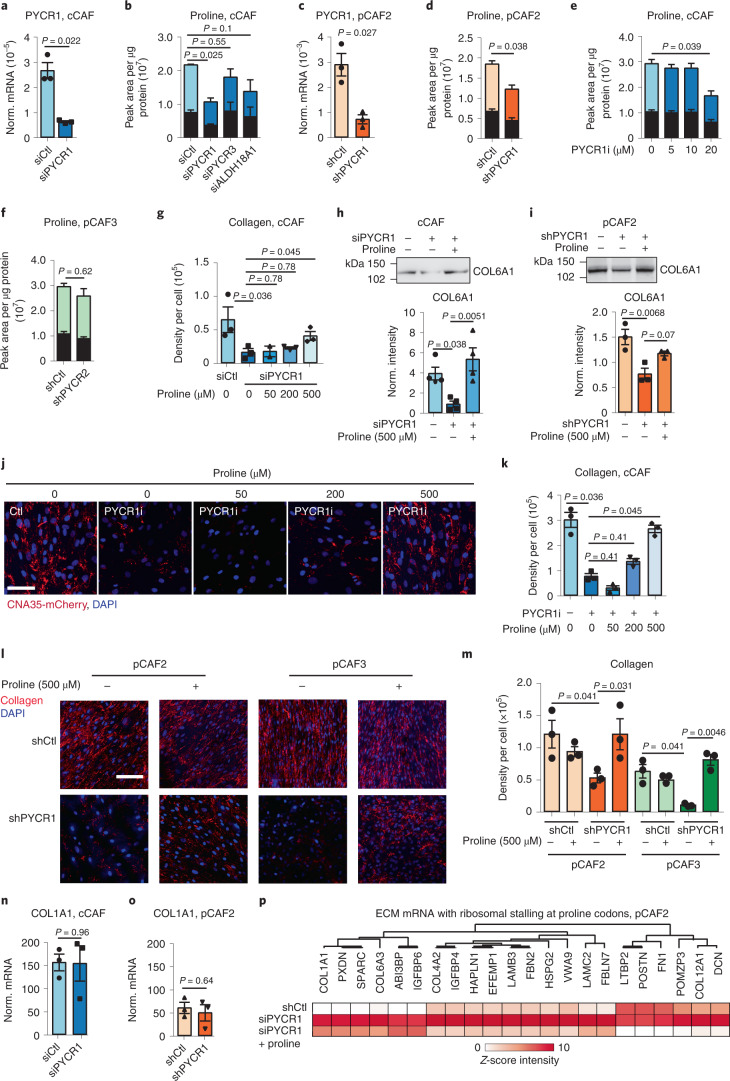
CAFs increase proline biosynthesis via PYCR1. **a**, *PYCR1* mRNA levels in cCAF transfected with siCtl/siPYCR1. *n* = 3 biological replicates. **b**, ^13^C-labelled (coloured) and unlabelled (black) proline in ^13^C-glutamine-labelled cCAFs transfected with siCtl, siPYCR1, siPYCR3 or siALDH18A1. *n* = 3 biological replicates. **c**, *PYCR1* mRNA levels in pCAF2 expressing shCtl or shPYCR1. *n* = 3 biological replicates. **d**, ^13^C-labelled (coloured) and unlabelled (black) proline in shCtl/shPYCR1 expressing pCAF2, labelled with ^13^C-glutamine. *n* = 3 biological replicates **e**, ^13^C-labelled (coloured) and unlabelled (black) proline measured by MS in cCAFs treated with 20 µM PYCR1i/dimethyl sulfoxide (DMSO) control, labelled with ^13^C-glutamine. *n* = 3 biological replicates. **f**, Total ^13^C-labelled (coloured) and unlabelled (black) proline in pCAF3 expressing shCtl/shPYCR2, labelled with ^13^C-glutamine. *n* = 3 biological replicates **g**, Quantification of collagen produced by cCAFs transfected with siCtl/siPYCR1 and 500 µM proline/PBS control. *n* = 3 biological replicates. **h**,**i**, Representative western blot and quantification for COL6A1 of decellularized ECM generated from cCAF (**h**) and pCAF (**i**) transfected with sh/siCtl or sh/siPYCR1 with 500 µM proline/PBS control. COL6A1 signal was normalized by Ponceau S staining (Extended Data Fig. 9). *n* = 3 biological replicates. **j**,**k**, Representative images (**j**) and quantification (**k**) of collagen produced by CAFs treated with 20 µM PYCR1i/DMSO control and 500 µM proline/PBS control. *n* = 3 biological replicates. **l**,**m**, Representative images (**l**) and quantification (**m**) of collagen produced by pCAF2 and pCAF3s expressing shCtl/shPYCR1 and with 500 µM proline/PBS control. *n* = 3 biological replicates. **n**, *COL1A1* mRNA levels in siCtl/siPYCR1 transfected cCAFs. *n* = 3 biological replicates. **o**, *COL1A1* mRNA in pCAF2 expressing shCtl/shPYCR1. *n* = 3 biological replicates. **p**, Diricore analysis of ribosome stalling on proline codons in ECM mRNAs of pCAF2 transfected with siCtl/siPYCR1 with 500 μM proline/PBS control. *n* = 3 biological replicates. Scale bar, 50 µm. Error bars indicate mean ± s.e.m. *P* values were calculated with two-tailed unpaired *t*-test with Welch’s correction (**a**, **c**, **d**, **f**, **n** and **o**), one-way ANOVA with Dunnett’s multiple comparison test (**b**, **e**, **h** and **i**) or Kruskal–Wallis with Dunn’s multiple comparison test (**g**, **k** and **m**). Source data

**Extended Data Fig. 3:**
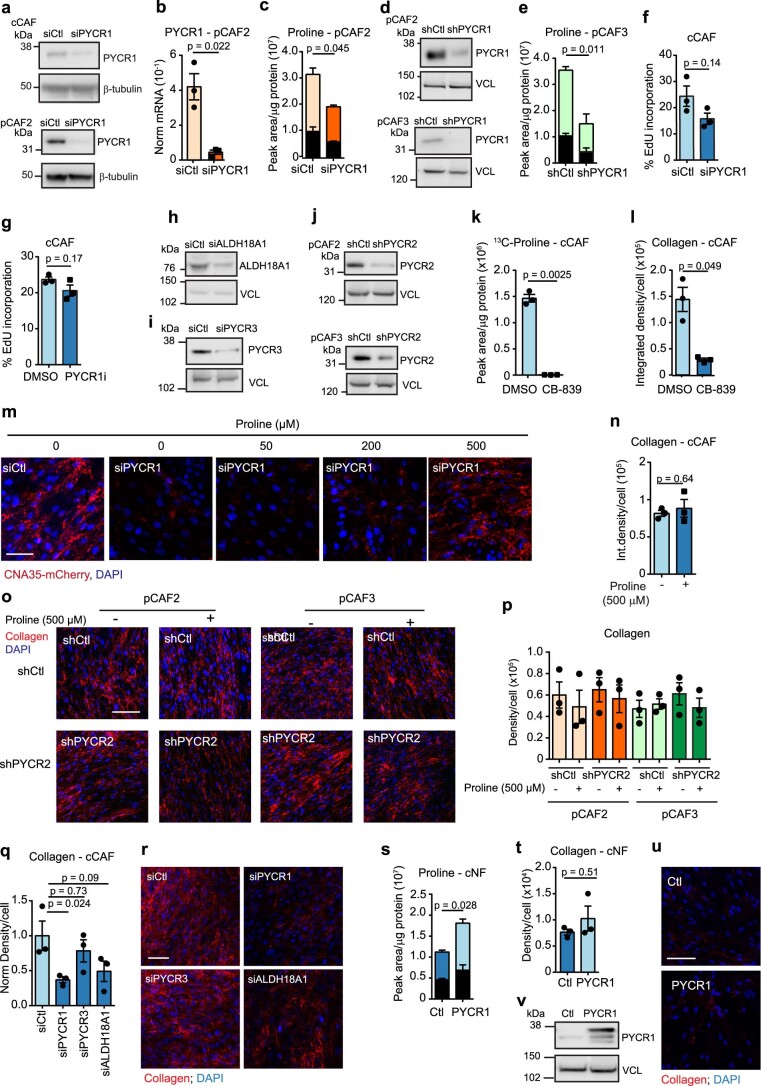
PYCR1 expression regulates proline synthesis and collagen production. **a**. Representative of 3 western blots of PYCR1 in cCAF and pCAF2 transfected with siCtl/siPYCR1. **b**. *PYCR1* mRNA in pCAF2 transfected with siCtl/siPYCR1. N = 3 biological replicates **c**. Total ^13^C-labelled (coloured) and unlabelled (black) proline in pCAF2 siCtl/siPYCR1, labelled with ^13^C_5_-Glutamine. N = 3 biological replicates **d**. Representative of 3 western blots of PYCR1 in shCtl and shPYCR1 pCAFs. **e**. ^13^C-labelled (coloured) and unlabelled (black) proline in pCAF3 shCtl/shPYCR1, labelled with ^13^C_5_-Glutamine,. N = 3 biological replicates. **f**. Proliferation of cCAF siCtl/siPYCR1. N = 3 biological replicates **g**. Proliferation of cCAF with 20 μM PYCR1i/DMSO control. N = 3 biological replicates. **h**. Representative of 3 western blots of cCAF siCtl/siALDH18A1. **i**. Representative of 2 western blots of cCAF siCtl/siPYCR3. **j**. Representative of 2 western blots of pCAF2/pCAF3 shCtl/shPYCR2. **k**. ^13^C-labelled proline in cCAFs with 50 nM CB-839/DMSO, labelled with ^13^C_5_-glutamine. N = 3 biological replicates. **l**. Collagen in cCAFs with 50 nM CB-839/DMSO. N = 3 biological replicates. **m**. Representative image of collagen produced by cCAF siCtl/siPYCR1 treated with 500 µM proline/PBS (quantification in Fig. 3g). **n**. Quantification of collagen produced by cCAFs treated with 500 μM proline/PBS. N = 3 biological replicates. **o, p**. Representative images (o) and quantification (p) of collagen from pCAF shCtl/shPYCR2 treated with 500 μM proline/PBS control. N = 3 biological replicates. **q, r**. Quantification (r) and representative images (q) of collagen produced by cCAF siCtl, siPYCR1 siPYCR3 or siALDH18A1. N = 3 biological replicates. **s**. Total ^13^C-labelled (coloured) and unlabelled (black) proline in cNFs transfected with pENTER-PYCR1 or pENTER-control, labelled with ^13^C_5_-Glutamine. N = 3 biological replicates. **t, u**. Quantification (t) and representative images (u) of collagen from cNFs transfected with pENTER-PYCR1 or pENTER-control. N = 3 biological replicates. **v**. Representative of 2 western blots of cNFs transfected with pENTER- PYCR1 pENTER-control. Error bars indicate mean ± SEM. p-values were calculated with 2-tailed, unpaired t-test with Welch’s correction (b, c, e, f, g, k, s), 2-tailed Mann-Whitney test (l, n, t) or Kruskal-Wallis with Dunn’s multiple comparison test (q). Scale bar = 50 µm. Source data

**Extended Data Fig. 4:**
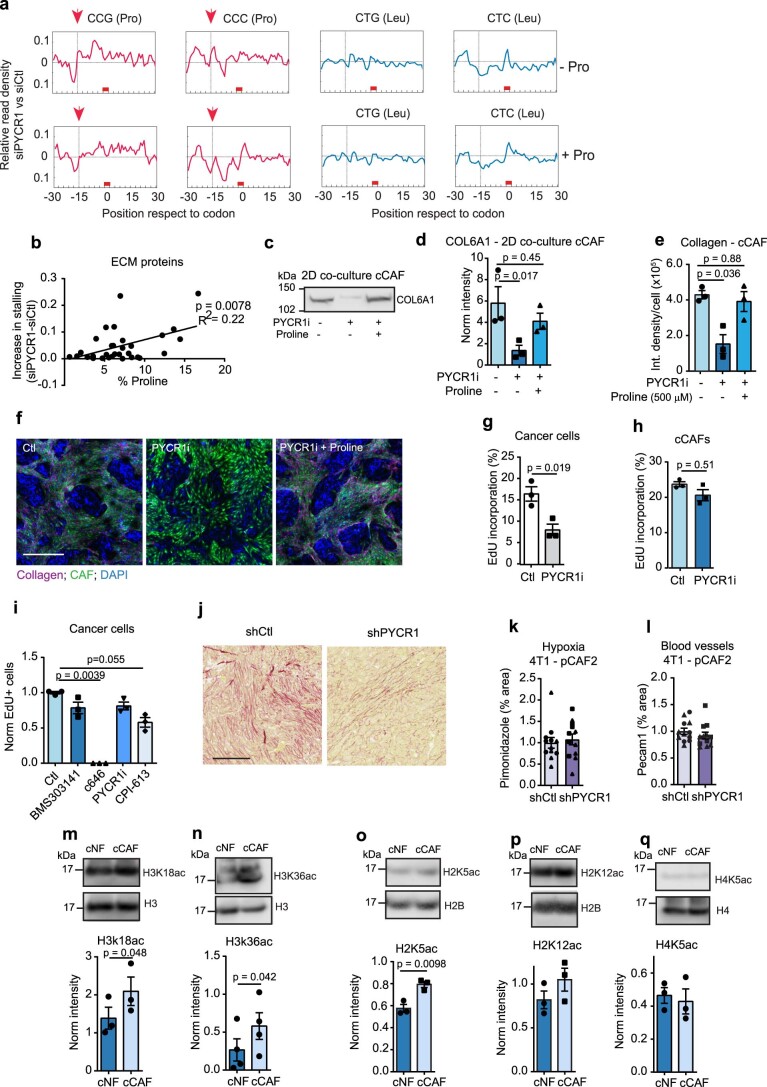
PYCR1 regulates collagen production *in vitro* and *in vivo*. **a**. Ribosome read density, at proline codons compared to Leucine codons in pCAF2 transfected with siCtl/siPYCR1. **b**. Correlation between % proline residues in ECM proteins and the increase in ribosome stalling in siPYCR1 compared to siCtl transfected pCAF2. **c, d**. Representative western blot (c) and quantification (d) of COL6A1 levels in ECM derived from 2D cCAF/cancer cell co-cultures with 20 μM PYCR1i/DMSO control, and 500 μM proline/PBS control. N = 3 biological replicates. **e, f**. Quantification (e) and representative images of collagen in 2D cCAF/cancer cell co-cultures with 20 μM PYCR1i/DMSO control, and 500 μM proline/PBS control. N = 3 biological replicates. Scale bar = 250 µm. **g, h**. Proliferation of cancer cells (g) and CAFs (h) in 2D cCAF/cancer cell co-cultures with 20 μM PYCR1i/DMSO control. N = 3 biological replicates **i**. EDU incorporation of breast cancer cells in mono-culture after treatment with DMSO control, 25 μM c646, 50 μM BMS303141, 20 μM PYCR1i or 50 μM CPI-613. N = 3 biological replicates. **j**. Representative images of Sirius Red staining in FFPE sections of 4T1 tumours co-transplanted with pCAF2 shCtl/shPYCR1 (quantification in Fig. 4s). Scale bar = 1000 µm. **k**. Quantification of Pimonidazole staining in FFPE sections of 4T1 tumours co-transplanted with pCAF2 shCtl/shPYCR1. N = 12 mice for each condition from two independent experiments. **l**. Quantification of Pecam1 staining in FFPE sections of tumours from (k). **m-q**. Quantification and representative western blots of acetylation levels at histone sites between cNFs and cCAFs. N = 3 or 4 biological replicates. Error bars indicate mean ± SEM. p values were calculated with a two-tailed Pearson r correlation (b), Kruskal-Wallis with Dunn’s multiple comparison test (d, e, i) or two-tailed t-test with Welch’s correction (g, h, m, n, o p, q). See Extended Data Fig. 9 for Ponceau-S staining of the blots used for COL6A1 in ECM, used as loading control. Source data

Inhibiting PYCR1 genetically with small interfering RNA (siRNA)/short hairpin RNA (shRNA), or pharmacologically with a small molecule inhibitor (PYCR1i),^35^ was sufficient to decrease ^13^C_5_-proline synthesis from ^13^C_5_-glutamine (Fig. 3a–e and Extended Data Fig. 3a–e), while not substantially affecting CAF proliferation (Extended Data Fig. 3f,g). Knockdown of PYCR2 and PYCR3 did not decrease proline synthesis, while ALDH18A1 knockdown decreased proline synthesis to a lesser extent than PYCR1 (Fig. 3b,f and Extended Data Fig. 3h,i). Similarly, inhibiting glutaminase (Fig. 1e) with the clinical compound CB-839 blocked proline synthesis and reduced collagen production in cCAF (Extended Data Fig. 3k,l). Genetic or pharmacological inhibition of PYCR1 in CAFs was sufficient to decrease collagen deposition in the ECM, as measured with the collagen probe CNA35-mCherry^36^ and western blot for COL6A1, which was almost fully rescued by providing CAFs with exogenous proline (Fig. 3g–m and Extended Data Fig. 3m,n). Notably, supplementing the culture medium with a supraphysiological concentration of proline^29^ was able to negate the effect of PYCR1 inhibition, while physiological levels of proline had only a minor impact. The addition of exogenous proline did not, however, increase collagen production in CAFs with no PYCR1 inhibition (Extended Data Fig. 3n), suggesting that endogenous PYCR1 activity produces sufficient proline for collagen production. Knockdown of PYCR2, PYCR3 and ALDH18A1 had either no effect or a lesser effect on collagen deposition (Extended Data Fig. 3o–r). Therefore, proline derived from PYCR1 is required to support collagen production, particularly when there is limited or physiological availability of exogenous proline. Further supporting this, the production of collagen proteins decreased upon silencing PYCR1, while *COL1A1* mRNA levels were unaltered (Fig. 3n,o). PYCR1 overexpression in NFs increased proline synthesis but had no effect on collagen production, supporting the theory that increased proline levels are only required for collagen synthesis in fibroblasts when there is increased expression of collagen genes (Extended Data Fig. 3s–v). Moreover, differential ribosome codon reading (Diricore) analysis^37^ showed that reduced levels of PYCR1 in CAFs induced ribosome stalling specifically at proline codons, which was rescued with the addition of exogenous proline (Extended Data Fig. 4a). The amount of stalling at proline codons for ECM proteins correlated with the proline content of the proteins (Extended Data Fig. 4b). Among the mRNAs that were affected by proline levels were several collagens, including *COL1A1* (Fig. 3p). Thus, a major function of PYCR1 in CAFs is to provide proline residues to maintain enhanced translation of collagen proteins.

### Targeting PYCR1 in CAFs reduces tumour growth and metastasis

**Fig. 4:**
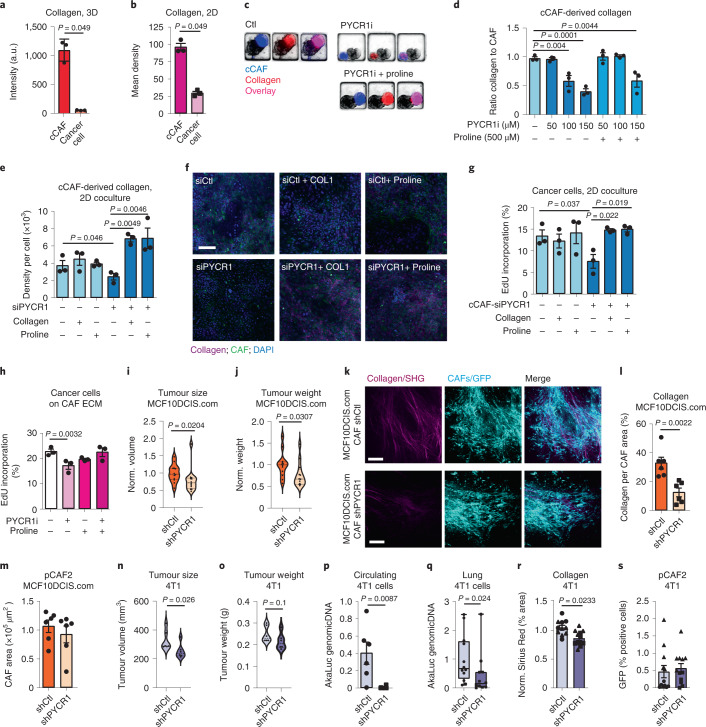
Stromal PYCR1 regulates collagen production and tumour progression in vitro and in vivo. **a**,**b**, Collagen quantification in 3D (**a**) and 2D cocultures (**b**). *n* = 3 biological replicates. **c**,**d**, Representative images (**c**) and quantification (**d**) of cCAF-derived collagen in 3D cocultures of CAFs and cancer cells treated with PYCR1i/DMSO control, and proline. *n* = 3 biological replicates. **e**,**f**, Representative images (**e**) and quantification (**f**) of cCAF-derived collagen in 2D cocultures of cCAFs siCtl/siPYCR1 and cancer cells, treated with 500 μM proline, 20 μg ml^−1^ collagen I or PBS control. *n* = 3 biological replicates. **g**, EdU incorporation of cancer cells in 2D cocultures with cCAFs siCtl/siPYCR1, treated with 500 μM proline, 20 μg ml^−1^ collagen I or PBS control. *n* = 3 biological replicates. **h**, EdU incorporation of cancer cells cultured on ECM from CAFs treated with 20 μM PYCR1i/DMSO control and 500 μM proline/PBS control. Control is the same as Figs. 5v and 6q. *n* = 3 biological replicates. **i**,**j**, Tumour volume (**i**) and weight (**j**) of MCF10DCIS.com xenografts cotransplanted with pCAF2 shCtl/shPYCR1. *n* = 12 mice for each condition from two experiments (six mice per experiment). **k**,**l**, Representative images (**k**) and quantification (**l**) of second harmonic generation signal surrounding shCtl/ shPYCR1 CAFs in tumours from **i**. *n* = 6 mice from each condition. **m**, Area of shCtl or shPYCR1 CAFs in tumours from **l**. **n**,**o**, Tumour volume (**n**) and weight (**o**) of 4T1 tumours cotransplanted with pCAF2 shCtl/shPYCR1. *n* = 6 mice for each condition. **p**, RT–qPCR quantification of circulating tumour cell DNA in blood from mice from **n**. **q**, RT–qPCR quantification of tumour cell DNA in lungs from mice with 4T1 tumours cotransplanted with pCAF2 shCtl/shPYCR1. *n* = 12 mice for each condition from two experiments (six mice per experiment). **r**, Sirius Red quantification in tumours from **q**. **s**, Quantification of transplanted CAFs in tumours from **q**. Error bars indicate mean ± s.e.m. *P* values were calculated with two-tailed Mann–Whitney test (**a**, **b**, **i**, **j** and **l**–**s**) or one-way ANOVA with Dunnett’s multiple comparison test (**d**, **e**, **g** and **h**). Scale bar, 50 µm. a.u., arbitrary units. Source data

As collagen influences tumour development, we asked whether targeting PYCR1 in CAFs could affect cancer cells, and cocultured cCAFs with a primary line of breast cancer cells either in a three-dimensional (3D) environment, as spheroids, in microfluidic devices,^38^ or in two dimensions. Imaging analysis of both cocultures showed that the majority of the collagen colocalized with the green fluorescent protein (GFP)-expressing CAFs (Fig. 4a,b), indicating that, similarly to in vivo^39^, CAFs are the major source of collagen. In 3D coculture, collagen production was reduced upon inhibition of PYCR1 and was rescued with exogenous proline (Fig. 4c,d). Similarly, inhibiting PYCR1 in two-dimensional (2D) coculture, pharmacologically and genetically in CAFs, strongly reduced collagen production, which was rescued with the addition of exogenous proline (Fig. 4e,f and Extended Data Fig. 4c–f). PYCR1 inhibition in CAFs in the coculture significantly reduced the proliferation of cancer cells but not CAFs (Fig. 4g and Extended Data Fig. 4g,h). Conversely, inhibiting PYCR1 in cancer cells in monoculture only modestly reduced proliferation (Extended Data Fig. 4i). Cancer cells also proliferated less when cultured on ECM derived from CAFs treated with PYCR1i (Fig. 4h). The treatment of PYCR1 inhibited CAFs with proline or soluble collagen I rescued the proliferation of cancer cells in coculture with the CAFs (Fig. 4g). Hence, we have revealed a potentially important role of PYCR1 in CAFs to support cancer cell growth by supporting enhanced collagen production.

Next, we targeted PYCR1 in CAFs in vivo. MCF10DCIS.com cells were cotransplanted with pCAFs expressing shCtl or shPYCR1 subcutaneously in Balb/c nude mice. Tumours containing CAFs shPYCR1 had reduced size and weight compared to those containing CAFs shCtl (Fig. 4i,j). Moreover, microscopy analysis of the tumours showed a marked decrease in fibrillar collagen deposited around pCAF shPYCR1 compared with pCAF shCtl, while the tumour area covered by CAF was unaffected (Fig. 4k–m). To assess whether targeting PYCR1 in CAFs impacts metastasis, we cotransplanted pCAFs shCtl or shPYCR1 with metastatic 4T1 breast cancer cells in NRMI nu/nu mice. Similarly to the MCF10DCIS.com xenograft model, the size and weight of the tumours containing CAFs shPYCR1 was reduced (Fig. 4n,o). The cotransplantation of pCAF shPYCR1 reduced the presence of circulating cancer cells in the blood (Fig. 4p), as well as the presence of metastatic cancer cells in the lungs (Fig. 4q). Tumours grown with pCAFs shPYCR1 contained less collagen than those with pCAFs shCtl (Fig. 4r and Extended Data Fig. 4j), while the amount of GFP-expressing CAFs, blood vessels and hypoxia were similar (Fig. 4s and Extended Data Fig. 4k,l). These data suggest that stromal PYCR1 may control metastatic cancer cell intravasation in the tumour blood vessels, at least in part, by the regulation of collagen deposition in the tumour stroma.

As a result, our data show that PYCR1 in mammary CAFs represents a stromal vulnerability for ECM production and can be targeted to reduce tumour growth and metastasis.

### CAFs have hyperacetylated histone 3

**Fig. 5:**
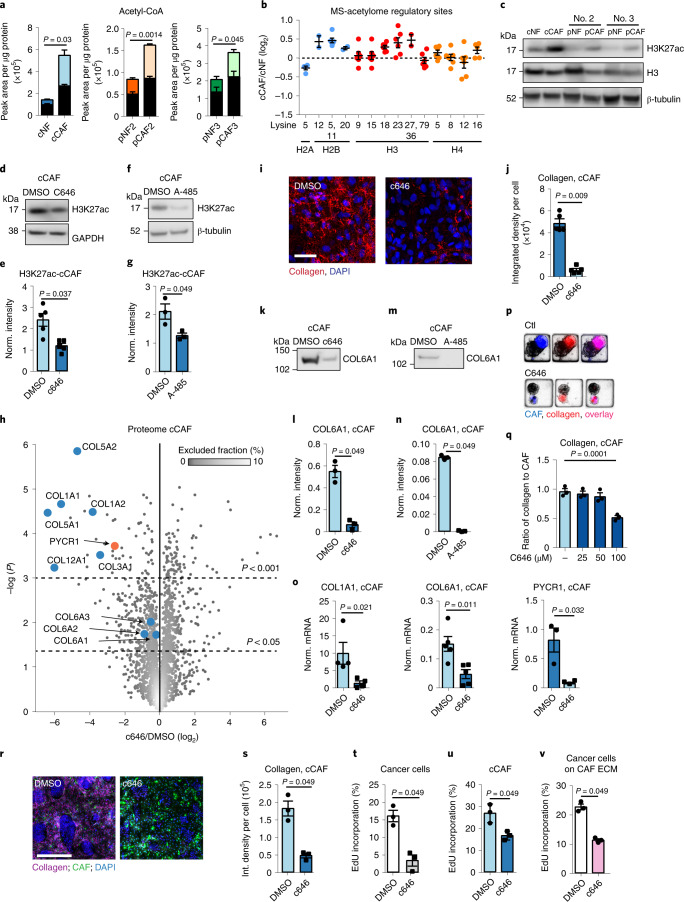
H3 acetylation regulates collagen production in CAFs. **a**, ^13^C-labelled (coloured) and unlabelled (black) acetyl-CoA in ^13^C-glucose labelled cCAFs/NFs. *n* = 3 biological replicates. **b**, SILAC ratios of regulatory histone acetylation sites identified in cNFs/cCAFs. *n* = 5 biological replicates. **c**, Representative of three western blots showing H3K27ac in NFs and CAFs. **d**,**e**, Representative western blot (**d**) and quantification (**e**) showing H3K27ac in cCAFs with 25 μM c646/DMSO control. *n* = 5 biological replicates. GAPDH, glyceraldehyde 3-phosphate dehydrogenase. **f**,**g**, Representative western blot (**f**) and quantification (**g**) showing H3K27ac in cCAFs with 3 μM A-485/DMSO control. *n* = 3 biological replicates. **h**, Volcano plot of cCAF proteome with 25 μM c646/DMSO control. *n* = 3 biological replicates. Plot coloured using density estimation function in MaxQuant. **i**,**j**, Representative images (**i**) and quantification (**j**) cCAF-derived collagen with 25 μM c646/DMSO control. *n* = 5 biological replicates. **k**,**l**, Representative western blot (**k**) and quantification (**l**) of COL6A1 in cCAF-derived ECM with 25 μM c646/DMSO control. *n* = 3 biological replicates. **m**,**n**, Representative western blot (**m**) and quantification (**n**) of COL6A1 cCAF-derived ECM with 3 μM A-485/DMSO control. *n* = 3 biological replicates. **o**, mRNA quantification in cCAFs with 25 μM c646/DMSO control. *n* ≥ 3 biological replicates. **p**,**q**, Representative images (**p**) and quantification (**q**) of collagen in 3D CAF/cancer cell cocultures with c646/DMSO control. *n* = 3 biological replicates. **r**,**s**, Representative images (*r*) and quantification (*s*) of collagen in 2D CAF/cancer cell cocultures with 25 μM c646/DMSO control. *n* = 3 biological replicates. **t**,**u**, Proliferation of cancer cells (**t**) and CAFs (**u**) in 2D coculture with 25 μM c646/DMSO control. *n* = 3 biological replicates. **v**, Proliferation of cancer cells seeded on ECM from CAFs treated with 25 μM c646/DMSO control. *n* = 3 biological replicates. Error bars indicate mean ± s.e.m. Significance was calculated with two-tailed, unpaired *t*-test with Welch’s correction (**a** and **o**), two-tailed Mann–Whitney test (**e**, **g**, **j**, **l**, **n**, **s**, **t**, **u** and **v**) or one-way ANOVA with Dunnett’s multiple comparison test (**q**). Scale bars, 50 µm (**i**) and 200 µm (**r**). See Extended Data Fig. 9 for Ponceau S staining of blots used for COL6A1 in ECM, used as loading control. Source data

**Extended Data Fig. 5:**
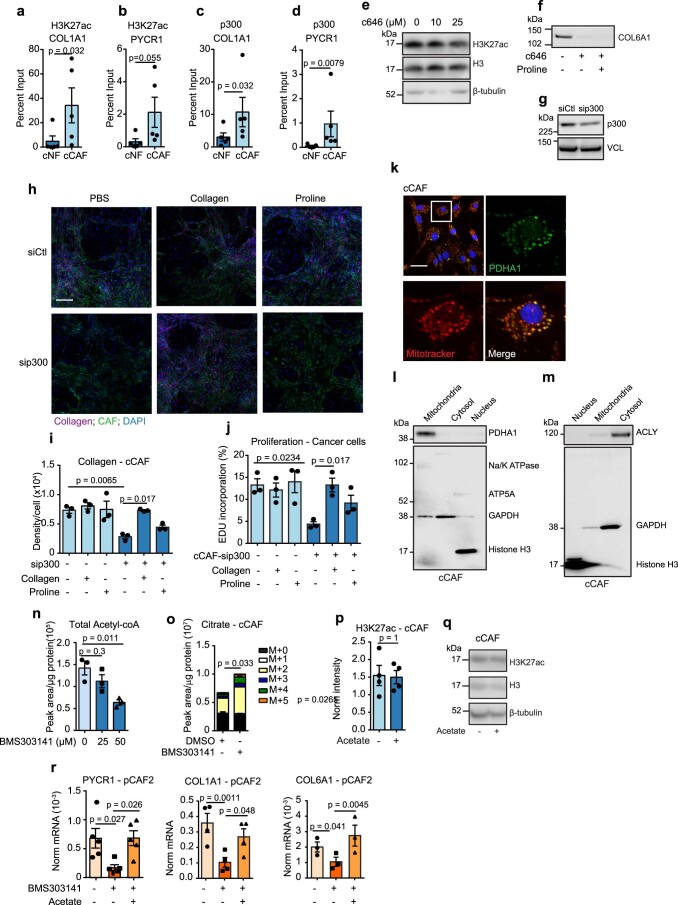
Histone acetylation regulates collagen production in CAFs. **a-d**. H3K27ac (a, b) and p300 (c, d) enrichment at *COL1A1* and *PYCR1* promoter regions in cNFs and cCAFs, measured by ChIP-qPCR. Signal was normalised to the input sample. N = 5 biological replicates. **e**. Representative of 4 western blots for H3K27ac in cCAFs treated with c646. **f**. Representative of 4 western blots of COL6A1 produced by cCAFs treated with 25 μM c646/DMSO control and 500 μM proline/PBS control. **g**. Representative of 3 western blots of EP300 in cCAFs transfected with siCtl/sip300. **h, i**. Representative images (i) and quantification (j) of collagen in co-cultures of cCAFs transfected with siCtl or sip300, and cancer cells. Cocultures were treated with 500 μM proline, 20 μg/ml collagen I, or PBS control. N = 3 biological replicates. Scale bar = 200 µm **j**. Proliferation of cancer cells in 2D cocultures of cCAFs transfected with siCtl /sip300, and cancer cells. Cocultures were treated with 500 μM proline, 20 μg/ml collagen I, or PBS as control. N = 3 biological replicates. **k**. Representative of >10 images of cCAFs showing co-localisation of PDHA1 with mitochondria (MitoTracker). Scale bar = 50 µm. **l, m**. Representative of 4 western blots of PDHA1 (l) and ACLY (m) in subcellular fractions of cCAFs. Na/K ATPase = membrane marker, ATP5A = mitochondrial marker, GAPDH = cytosolic marker, H3 = nuclear marker. **n**. Total acetyl-CoA in cCAF treated with DMSO control, or BMS303141 at the indicated concentrations. N = 3 biological replicates. **o**. ^13^C-glucose-labelled (coloured) and unlabelled (black) citrate in cCAFs with 50 µM BMS303141/DMSO control. N = 3 biological replicates. **p, q**. Representative western blot (p) and quantification (q) of H3K27 levels in cCAFs with 1 mM acetate/PBS control. N = 3 biological replicates. **r**. mRNA levels in pCAF2 with 50 µM BMS303141 or DMSO control and acetate or PBS control. N = 3-5 biological replicates. Error bars indicate mean ± SEM. p-values were calculated with 2-tailed Mann-Whitney test (a-d, n, p), 2-tailed unpaired t-test with Welch’s correction (p) Kruskal-Wallis with Dunn’s multiple comparisons test (i,j,o) or 1-way ANOVA with Dunnett’s multiple comparisons test (r). Source data

We next sought to determine the signalling that supports collagen production and PYCR1 expression in CAFs. Our MS-metabolomic analyses showed that levels of acetyl-CoA were consistently higher in CAFs than NFs (Fig. 5a and Extended Data Fig. 1f). Acetyl-CoA is a signalling molecule and acetyl donor for acetylation of proteins and histones and is, therefore, a central epigenetic regulator^40,41^. MS-based global acetylation analysis of cCAF and cNF showed histone 3 was more acetylated in CAFs at sites that are substrates of the epigenetic regulator histone acetyl-transferase EP300 (Fig. 5b and Supplementary Data 5)^42^. We confirmed H3K27, H3K18 and H3K36 hyperacetylation in CAFs by western blot analysis (Fig. 5c and Extended Data Fig. 4m,n). In accordance with the MS-acetylation data, H2BK5 and H2BK12 were also more acetylated in cCAFs, whereas H4K5 was not (Extended Data Fig. 4o–q). We focused on H3K27 because its hyperacetylation is an established marker of enhanced transcription^43^, and increased levels of acetyl-CoA has been shown to promote its acetylation through EP300 (ref. ^44^). Furthermore, the recruitment of EP300 and hyperacetylation of histone 3 at enhancers of profibrotic genes are a landmark event in fibrosis and TGFβ-stimulated gene expression^45,46^. Using a ChIP–quantitative PCR (qPCR) approach, we found that both H3K27ac and EP300 were enriched at *COL1A1* and *PYCR1* promoters in cCAFs compared to cNFs (Extended Data Fig. 5a–d). Based on these grounds, we investigated the link between EP300, acetyl-CoA levels and histone acetylation with collagen production in CAFs.

### EP300 supports collagen production in CAFs

Treating cCAFs with the EP300 inhibitors c646 or A-485 reduced H3K27 acetylation (Fig. 5d–g and Extended Data Fig. 5e). To determine whether this reduction in histone acetylation corresponded to a regulation of collagen production, we performed MS-proteomic analysis of cCAFs after 24 h treatment with c646 (Supplementary Data 6). Strikingly, collagens were among the most highly downregulated proteins by c646 treatment and PYCR1 was also strongly downregulated (Fig. 5h). By microscopy and western blot we further confirmed that EP300 inhibition reduced collagen deposition (Fig. 5i–n), which was not rescued by exogenous proline (Extended Data Fig. 5f). This demonstrated again that proline supports increased collagen production only when there is increased collagen gene expression. Moreover, inhibiting EP300 decreased expression of collagens and *PYCR1* at the transcriptional level (Fig. 5o), suggesting that EP300 epigenetically regulates collagen production. This co-regulation of *PYCR1* and collagens further supports our evidence that proline synthesis is upregulated to support collagen production in CAFs.

CAFs deposited less collagen when treated with c646 in 3D coculture with cancer cells (Fig. 5p,q). Similarly in 2D coculture, inhibiting EP300 either pharmacologically or genetically in CAFs inhibited collagen deposition and reduced cancer cell and CAF proliferation (Fig. 5r–u and Extended Data Fig. 5g–j). Inhibiting EP300 also blocked cancer cell proliferation when in monoculture (Fig. 5t and Extended Data Fig. 4i), in line with previous reports^42^. Cancer cell proliferation was less affected upon c646 treatment in the coculture than in the monoculture (Extended Data Fig. 4c), suggesting that CAFs may have some protective effects. However, cancer cell proliferation was also inhibited when cultured on ECM derived from CAFs treated with c646 (Fig. 5v) or cocultured with CAFs transfected with siEP300 (Extended Data Fig. 5j). Cancer cell proliferation was rescued by soluble collagen I, but not by exogenous proline (Extended Data Fig. 5j), in line with our data that shows that proline does not rescue collagen production in EP300 inhibited CAFs. Therefore, it is clear that changes in collagen production upon EP300 silencing in CAFs play a key role in the observed phenotype. Hence, EP300 activity regulates collagen production in CAFs and can be targeted to reduce cancer cell growth.

### Acetyl-CoA levels regulate CAF collagen production

**Fig. 6:**
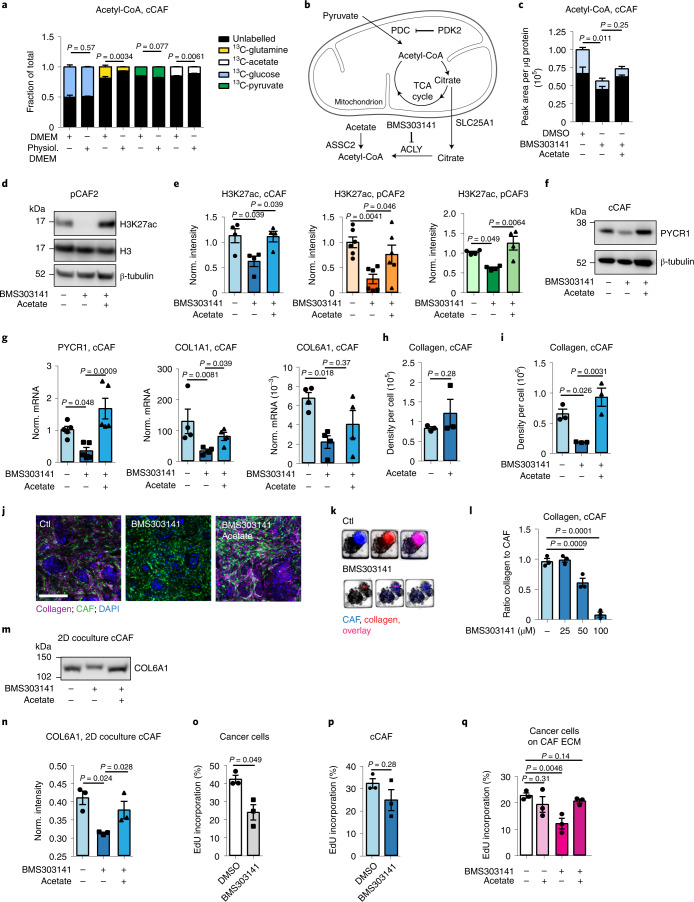
Nucleo-cytosolic acetyl-CoA regulates collagen production. **a**, ^13^C-labelled metabolite incorporation in cCAF cultured in DMEM or physiol. DMEM (5 mM glucose, 0.65 mM glutamine, 100 µM pyruvate and 100 µM acetate). **b**, Acetyl-CoA production/export from mitochondria. **c**, Unlabelled (black) and ^13^C-labelled (coloured) acetyl-CoA in ^13^C-glucose labelled cCAFs with 50 μM BMS303141/DMSO control and 1 mM acetate/PBS control. **d**,**e**, Representative western blot (**d**) and quantification (**e**) showing H3K27ac in CAFs with 50 μM BMS303141/DMSO control and 1 mM acetate/PBS control. *n* = 4–6 biological replicates. **f**, Representative western blot of PYCR1 in cCAFs with 50 μM BMS303141/DMSO control and 1 mM acetate/PBS control (1 of 3). **g**, mRNA quantification in cCAFs with 50 μM BMS303141/DMSO control and 1 mM acetate/PBS control. *n* = 3 biological replicates. **h**, Collagen quantification with 1 mM acetate/PBS control. *n* = 3 biological replicates. **i**,**j**, Representative images (**j**) and quantification (**i**) of collagen in 2D cCAF/cancer cell cocultures with 50 μM BMS303141/DMSO control and 1 mM acetate/PBS control. *n* = 3 biological replicates. **k**,**l**, Representative images (**k**) and quantification (**l**) of collagen in 3D cCAF/cancer cell cocultures with BMS303141/DMSO control and 1 mM acetate/PBS control. *n* = 3 biological replicates. **m**,**n**, Representative western blot (**m**) and quantification (**n**) of COL6A1 in ECM from cCAFs with 50 μM BMS303141/DMSO control and 1 mM acetate/PBS control. *n* = 3 biological replicates. **o**,**p**, Proliferation of cancer cells (**o**) and CAFs (**p**) in cocultures treated with 50 μM BMS303141/DMSO control. *n* = 3 biological replicates. **q**, Proliferation of cancer cells seeded on ECM from cCAFs treated with 50 μM BMS303141/DMSO control and 1 mM acetate/PBS control. *n* = 3 biological replicates. Error bars indicate mean ± s.e.m. *P* values were calculated with two-tailed, unpaired *t*-test with Welch’s correction (**a**), two-tailed Mann–Whitney test (**o** and **p**), one-way ANOVA with Dunnett’s multiple comparison test (**c**, **g**, **i**, **l** and **n**) or Kruskal–Wallis with Dunn’s multiple comparison test (**e** and **q**). Scale bar, 200 µm. See Extended Data Fig. 9 for Ponceau S staining of blots used for COL6A1 in ECM, used as loading control. Source data

Since EP300 activity is dependent on nucleo-cytosolic acetyl-CoA levels^44,47^, we determined the source of acetyl-CoA in CAFs. Tracing experiments with heavy carbon-labelled metabolites showed that glucose contributed around 50% of the total acetyl-CoA, while acetate, glutamine and pyruvate contributed to a lesser extent, with minor differences depending on whether cells were cultured in DMEM or physiol. DMEM, which contains physiological levels of glucose (5 mM), glutamine (0.65 mM), acetate (100 µM) and pyruvate (100 µM) (Figs. 5a and 6a). As a result, we conclude that in cultured CAFs most of the acetyl-CoA is synthesized from glucose-derived pyruvate via the mitochondrial pyruvate dehydrogenase complex (PDC) (Fig. 6b). There is evidence that PDC can translocate into the nucleus to synthesize acetyl-CoA^48^, but we could not detect the regulatory subunit pyruvate dehydrogenase E1 component alpha A1 (PDHA1) in the nucleus by immunofluorescence or western blot analysis (Extended Data Fig. 5k,l). Acetyl‐CoA efflux from the mitochondria to the cytosol occurs through citrate and is converted back to acetyl-CoA in the cytosol or nucleus by ATP citrate lyase (ACLY) (Fig. 6b). In cCAFs, ACLY was only detected in the cytoplasm (Extended Data Fig. 5m). However, small molecules such as acetyl-CoA can freely enter the nucleus through nuclear pores. To assess whether levels of nucleo-cytosolic acetyl-CoA influence H3K27 acetylation in CAFs, we used a previously established model, in which nucleo-cytosolic acetyl-CoA is reduced by inhibiting ACLY, and replenished by adding exogenous acetate. Acetate is converted to acetyl-CoA by acetyl-coenzyme A synthetase 2 (ACSS2)^44^ (Fig. 6b). Treatment with the inhibitor BMS3031414 effectively inhibited ACLY, as it induced accumulation of ^13^C_2_-citrate-derived from ^13^C_6_-glucose, while reducing ^13^C_2_-labelled acetyl-CoA, and total acetyl-CoA levels were restored with acetate supplementation (Fig. 6c and Extended Data Fig. 5n,o). Inhibiting ACLY consistently reduced H3K27 acetylation, which was recovered with exogenous acetate (Fig. 6d,e). Stimulating untreated CAFs with acetate did not increase histone acetylation or collagen production (Fig. 6h and Extended Data Fig. 5p,q), suggesting that while acetate/ACSS2 promote H3K27 acetylation when acetyl-CoA is limited, they do not drive the increase in histone acetylation in CAFs. Inhibiting ACLY in cCAFs (Fig. 6f,g) and pCAFs (Extended Data Fig. 5r) reduced expression of *PYCR1*, *COL1A1* and *COL6A1*, PYCR1 protein levels and collagen deposition (Fig. 6f,g), which were rescued with acetate, supporting the premise that acetyl-CoA levels epigenetically control collagen production. BMS303141 treatment also reduced collagen production when CAFs were cocultured with breast cancer cells in 2D and 3D (Fig. 6i–n). Similarly to inhibiting PYCR1, BMS303141 opposed cancer cell proliferation, which was restored by acetate, while having no significant impact on CAF growth (Fig. 6o,p). BMS303141 treatment had minimal impact on the proliferation of cancer cells cultured alone (Extended Data Fig. 4c). Moreover, cancer cells proliferated less on ECM derived from BMS303141-treated CAFs (Fig. 6q). Thus, nucleo-cytosolic levels of acetyl-CoA in CAFs influence collagen production, which supports cancer cell growth.

### PDK2 is a key regulator of acetyl-CoA levels in CAFs

**Fig. 7:**
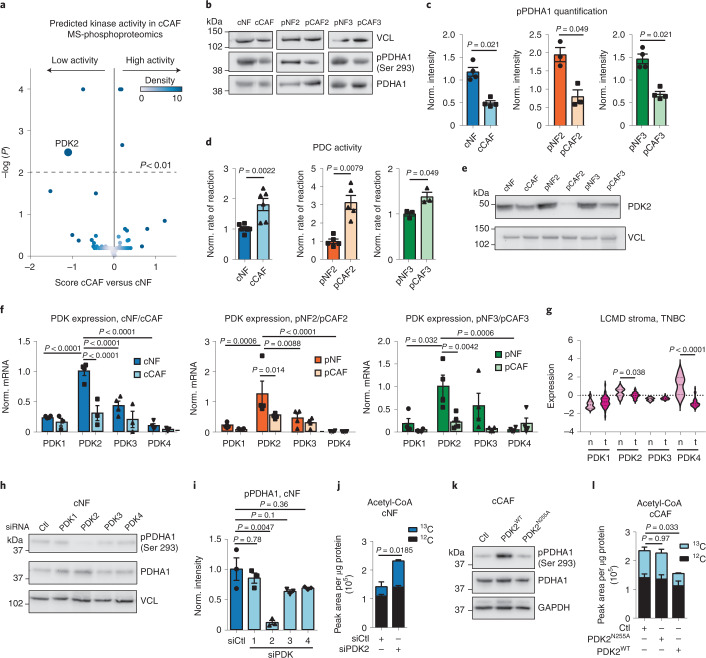
PDK2 regulates PDH activity and acetyl-CoA production in CAFs. **a**, Predicted kinase activity in cCAFs compared to cNFs based on the modelling of their MS-based phosphoproteomic data. **b**,**c**, Representative western blots (**b**) and quantification (**c**) showing PDHA1 phosphorylation levels at the regulatory site S293 in mammary NFs and CAFs. VCL was used as a loading control. *n* = 3 or 4 biological replicates. **d**, Pyruvate dehydrogenase activity of NFs and CAFs measured as the rate of NAD+ reduction in vitro. *n* = 3–6 biological replicates. **e**, Representative western blot showing PDK2 levels in paired mammary NFs and CAFs. VCL was used as a loading control. **f**, *PDK1–4* expression in mammary NFs and CAFs in culture, measured by qPCR and normalized to 18S expression. *n* = 4 biological replicates. **g**, *PDK1–4* mRNA expression in LCMD sections of normal and TNBC-associated stroma from Saleh et al.^31^. **h**,**i**, Representative western blot (**h**) and quantification (**i**) showing PDHA1 phosphorylation levels in cNFs transfected with siCtl or siPDK1–4. *n* = 3 biological replicates. VCL was used as a loading control. **j**, Intracellular acetyl-CoA unlabelled (black) and ^13^C_2_-labelled (coloured) from ^13^C_6_-glucose measured by MS in cNFs transfected with siCtl or siPDK2. *n* = 3 biological replicates. **k**, Representative western blot showing PDHA1 phosphorylation levels in cCAFs transfected with empty vector, pGC-PDK2^N255A^ or pGC-PDK2^WT^. **l**, ^13^C_2_-labelled (coloured bar) and unlabelled (black bar) acetyl-CoA measured by MS in cCAFs transfected with empty vector, pGC-PDK2^N255A^ or pGC-PDK2^WT^ and labelled with ^13^C_6_-glucose. *n* = 3 biological replicates. Error bars indicate mean ± s.e.m. *P* values were calculated with two-tailed, unpaired *t*-test with Welch’s correction (**g** and **j**), two-tailed Mann–Whitney test (**c** and **d**), two-way ANOVA with Tukey’s multiple comparisons test (**f**), one-way ANOVA with Dunnett’s multiple comparison test (**l**) or Kruskal–Wallis with Dunn’s multiple comparison test (**i**). Source data

**Extended Data Fig. 6:**
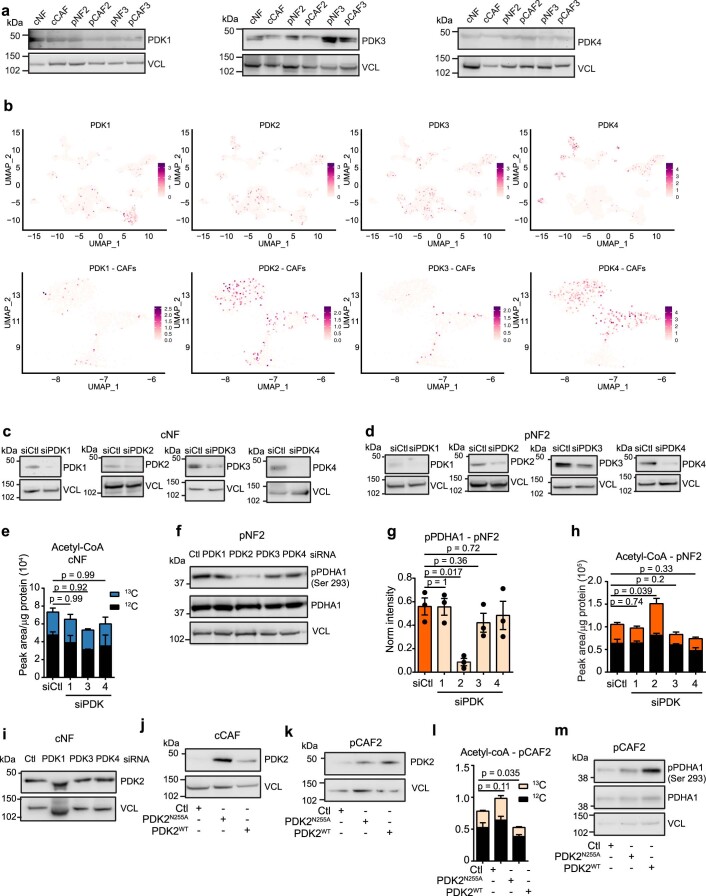
PDK2 regulates acetyl-CoA production in patient derived fibroblasts. **a**. Representative of 2 western blots showing levels of PDK1, PDK3 and PDK4 in mammary NFs and CAFs. **b**. UMAP visualisation of stromal, immune and cancer cells (top) or CAF-only (bottom) aligned using canonical correlation analysis in Seurat. Cell types defined as in Wu et al. **c,d**. Representative of 3 western blots showing expression of PDK1-4 after transfection with siCtl or the respective siRNA in cNF (c) and pNF2 (d). **e**. ^13^C-labelled (coloured) and unlabelled (black) acetyl-CoA in cNF2 transfected with siCtl or siPDK1,3,4, labelled with ^13^C_6_-glucose. N = 3 biological replicates. **f,g**. Representative western blot (f) and quantification (g) showing pPDHA1 levels in pNF2 transfected with siCtl /siPDK1-4. N = 3 biological replicates. **h**. ^13^C-labelled (coloured) and unlabelled (black) acetyl-CoA in pNF2 transfected with siCtl/siPDK1-4, labelled with ^13^C_6_-glucose. N = 3 biological replicates. **i**. Representative of 2 western blots showing PDK2 levels in cNF transfected with siCtl, siPDK1, siPDK3, or siPDK4. **j,k**. Representative of 2 western blots showing the levels of overexpression of PDK2 wild type (PDK2^WT^) or the enzymatically inactive mutant form (PDK2^N255A^) in cCAF (j) and pCAF2 (k) after transfection with empty vector (Ctl), pGC-PDK2^N255A^/pGC-PDK2^WT^. **l**. ^13^C-labelled (coloured bar) and unlabelled (black bar) acetyl-CoA in pCAF2 transfected with empty vector/pGC-PDK2^N255A^/pGC-PDK2^WT^ and labelled with ^13^C_6_-glucose. N = 3 biological replicates. **m**. Representative of 3 western blots showing PDHA1 phosphorylation levels in pCAF2 after transfection with empty vector/pGC-PDK2^N255A^/pGC-PDK2^WT^. Error bars indicate mean ± SEM. p-values were calculated with 1-way ANOVA with Dunnett’s multiple comparisons test (e, h, l) or Kruskal-Wallis with Dunn’s multiple comparisons test (g) VCL = vinculin. Source data

Our MS-metabolomic data indicated that PDC is the major source of increased acetyl-CoA in CAFs (Figs. 5a and 6a). PDC activity is negatively regulated by pyruvate dehydrogenase kinase proteins (PDK1–4), which phosphorylate PDHA1. Intriguingly, computational analysis of our phosphoproteomic analysis of cCAF and cNF (Supplementary Data 7) predicted PDK2 to be the most de-activated kinase in cCAF (Fig. 7a and Supplementary Data 8). Concordantly, the phosphorylation level of PDHA1 at the regulatory site S293 was consistently lower in CAFs than NFs (Fig. 7b,c and Supplementary Data 7), PDC activity was higher in CAFs and CAFs had lower levels of PDK2 than NFs (Fig. 7d,e). When we assessed PDK mRNA levels, *PDK2* was the most highly expressed in NFs and strongly downregulated in CAFs (Fig. 7f). CAFs also expressed low levels of *PDK1*, *PDK3* and *PDK4*, and there was no consistent differences in their expression between CAFs and NFs (Fig. 7f and Extended Data Fig. 6a). Gene expression data of microdissected stroma from normal breast and TNBC^31^ showed significant downregulation of *PDK2* and low levels of *PDK1*, *PDK3* and *PDK4* in the tumour stroma (Fig. 7g) similarly to our CAFs. Re-analysis of available TNBC scRNA-seq data^3^ further showed that few CAFs expressed PDKs if they had high levels of collagen (Extended Data Figs. 2b and 6b). PDK4 was expressed by perivascular and endothelial cells, which may explain the high PDK4 levels measured in normal breast stroma (Fig. 7g).

Next, we silenced each of the PDKs in NFs. While all were efficiently silenced (Extended Data Fig. 6c,d), only siPDK2 reduced PDHA1 phosphorylation and PDC-derived ^13^C_2_-acetyl-CoA (Fig. 6h–j and Extended Data Fig. 6e–h). The lack of effect of PDK1, PDK3 and PDK4 silencing on PDHA1 phosphorylation was not due to any compensation by *PDK2* expression, as PDK2 protein levels were unaffected (Extended Data Fig. 6i). Overexpression of PDK2 wild type (PDK2^WT^), but not a mutant enzymatically inactive form^49^ (PDK2^N255A^) (Extended Data Fig. 6j,k), increased PDHA1 phosphorylation and the production of acetyl-CoA in cCAFs (Fig. 6k,l) and pCAFs (Extended Data Fig. 6l,m). Hence, PDK2 is the most expressed PDK in mammary fibroblasts in vitro and a major regulator of PDC activity and acetyl-CoA levels.

### PDC activity promotes collagen production in fibroblasts

**Fig. 8:**
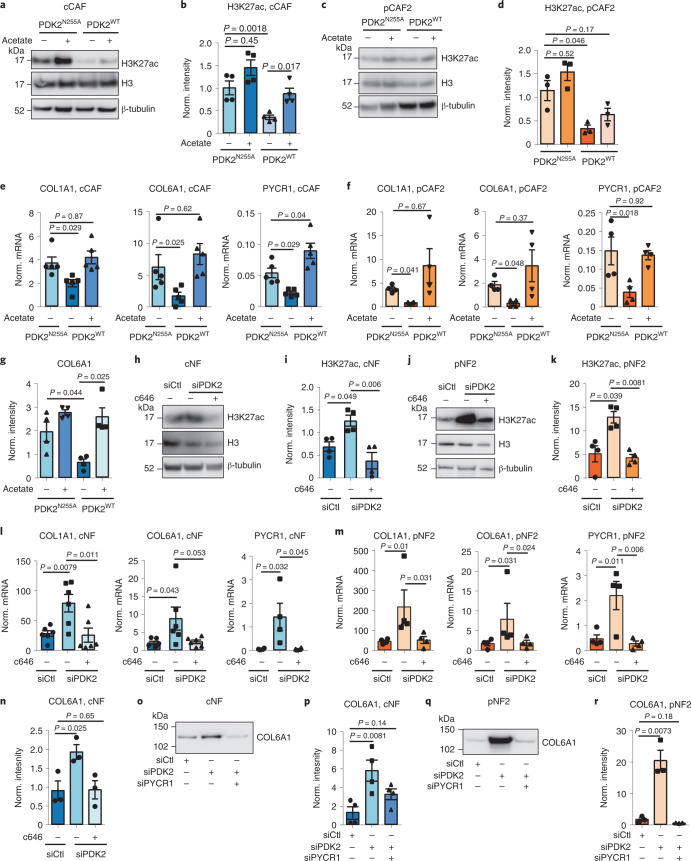
PDH activation regulates collagen production in CAFs. **a**,**b**, Representative western blot (**a**) and quantification (**b**) of H3K27ac in cCAFs transfected with pGC-PDK2^N255A^/pGC-PDK2^WT^ with 1 mM acetate/PBS control. *n* = 4 biological replicates. **c**,**d**, Representative western blot (**c**) and quantification (**d**) of H3K27ac in pCAF2 transfected with pGC-PDK2^N255A^ or pGC-PDK2^WT^ with 1 mM acetate/PBS control. *n* = 3 biological replicates. **e**, mRNA quantification in cCAFs transfected with pGC-PDK2^N255A^/pGC-PDK2^WT^. *n* = 3–5 biological replicates. **f**, mRNA expression of *COL1A1*, *COL6A1* and *PYCR1* in pCAF2 transfected with pGC-PDK2^N255A^/pGC-PDK2^WT^ with 1 mM acetate/PBS control. *n* = 3 biological replicates. **g**, Quantification of COL6A1 levels in ECM from cCAFs transfected with pGC-PDK2^N255A^/pGC-PDK2^WT^ and with 1 mM acetate/PBS control. *n* = 3 biological replicates. **h**,**i**, Representative western blot (**h**) and quantification (**i**) of H3K27ac in cNFs transfected with siCtl/siPDK2 with c646/DMSO control. *n* = 4 biological replicates. **j**,**k**, Representative western blot (**j**) and quantification (**k**) of H3K27ac in pNF2 transfected with siCtl/siPDK2 with c646/DMSO control. *n* = 4 biological replicates. β-tubulin was used as a loading control. **l**,**m**, mRNA quantification in cNFs (**l**) and pNF2 (**m**) transfected with siCtl/siPDK2 with c646/DMSO control. *n* = 6 or 4 biological replicates. **n**, Quantification of COL6A1 in decellularized ECM derived from pNF transfected with siCtl/siPDK2 with c646/DMSO control. *n* = 3 biological replicates. **o**,**p**, Representative western blot (**o**) and quantification (**p**) of COL6A1 in decellularized ECM derived from cNFs transfected with siCtl, siPDK2 or siPDK2 + siPYCR1. *n* = 3 biological replicates. **q**,**r**, Representative western blot (**q**) and quantification (**r**) of COL6A1 in ECM derived from pNF2 transfected with siCtl, siPDK2 or siPDK2 + siPYCR1. *n* = 3 biological replicates. Error bars indicate mean ± s.e.m. *P* values were calculated with one-way ANOVA with Dunnett’s multiple comparison test (**e**, **f**, **l** and **m**) or Kruskal–Wallis with Dunn’s multiple comparison test (**b**, **d**, **g**, **I**, **k**, **n**, **p** and **r**). See Extended Data Fig. 9 for Ponceau S staining of blots used for COL6A1 in ECM, used as loading control. Source data

**Extended Data Fig. 7:**
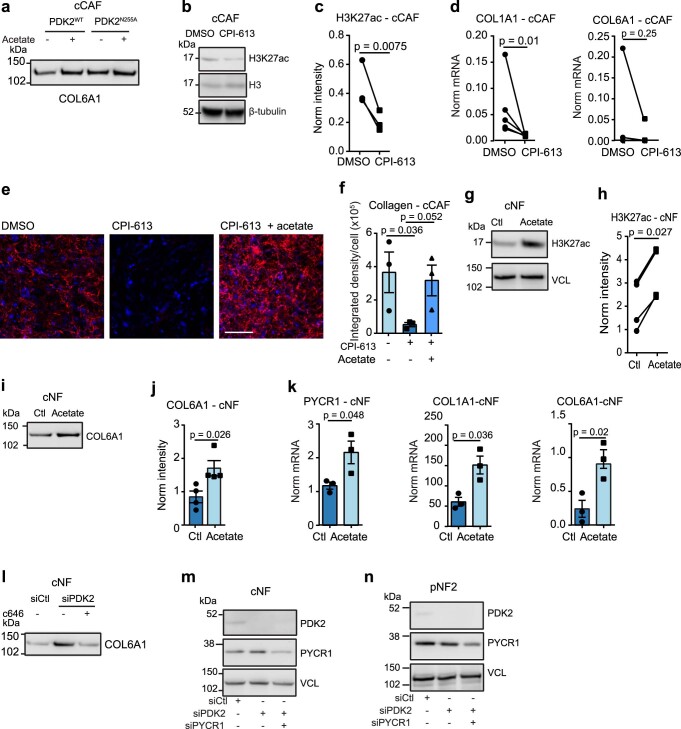
PDH inhibition regulates collagen production. **a**. Representative western blot (quantification in Fig. 8g) showing COL6A1 levels in ECM derived from cCAF transfected with pGC-PDK2^N255A^/pGC-PDK2^WT^ and treated with acetate or PBS control. **b, c**. Representative western blot (b) and quantification (c) of H3K27ac levels in cCAF with 50 µM CPI-613 or DMSO control. N = 3 biological replicates. **d**. Expression of *COL1A1 and COL6A1* in cCAF with 50 µM CPI-613 or DMSO control, normalised to 18S expression. N = 3 biological replicates. **e, f**. Representative confocal microscopy images (e) and quantification (f) of collagen produced by cCAF with 50 µM CPI-613 or DMSO control and 1 mM acetate or PBS, as control. N = 3 biological replicates. Scale bar = 100 µm. **g, h**. Representative western blot (g) and quantification (h) of H3K27ac levels in cNF treated with acetate or PBS control. N = 3 biological replicates. **i, j**. Representative western blot (i) and quantification (j) of COL6A1 levels in decellularised ECM derived from cNF treated with 1 mM acetate or PBS control. N = 3 biological replicates. **k**. Expression of *PYCR1, COL1A1 and COL6A1* in cNF with 1 mM acetate or PBS control, normalised to 18S expression. N = 3 biological replicates. **l**. Representative western blot of COL6A1 levels in ECM derived from cNF transfected with siCtl/siPDK2 with 25 µM c646/DMSO control (quantification in Fig. 8m). **m, n**. Representative of 2 western blots showing PDK2 and PYCR1 levels in cNF (m) and pNF2 (n) transfected with siCtl, siPDK2 or siPDK2 and siPYCR1. Error bars indicate mean ± SEM. p-values were calculated with 2-tailed, paired t-test (c-e, h), 2-tailed, unpaired t-test (j, k) or Kruskal Wallis with Dunn’s multiple comparisons test (f). Scale bar = 50 µm. VCL = vinculin. See Extended Data Fig. 9 for Ponceau-S staining of blots used for COL6A1 in the ECM, used as a loading control. Source data

Next, we determined whether PDC activity controls collagen and proline production. Overexpression of PDK2 in CAFs to inactivate PDC reduced H3K27 acetylation, as well as *COL1A1*, *COL6A1* and *PYCR1* expression and collagen deposition in the ECM (Fig. 8a–g), and this was counteracted by exogenous acetate (Fig. 8a–g and Extended Data Fig. 7a). Inhibiting PDC with the clinical compound CPI-613 gave similar results (Extended Data Fig. 7b–f). Exogenous acetate was also able to induce H3K27 acetylation, expression of collagen and *PYCR1*, and collagen deposition in the cNF (Extended Data Fig. 7g–k); therefore, increasing acetyl-CoA levels alone is sufficient to induce collagen production in NFs. Silencing PDK2 in cNF or pNF to increase PDC activity also enhanced H3K27 acetylation, *COL1A1*, *COL6A1* and *PYCR1* expression, and collagen deposition in the ECM (Fig. 8h–o and Extended Data Fig. 7l). These effects were negated when inhibiting EP300 with c646 (Fig. 8h–o and Extended Data Fig. 7l).

Finally, we assessed the requirement for PYCR1 to support increased collagen production following increased PDC activity. We silenced PDK2 in NFs, alone or together with PYCR1 (Extended Data Fig. 7m,n), and monitored collagen production. Reducing PYCR1 levels in NFs silenced for PDK2 was sufficient to inhibit PDC-induced collagen production (Fig. 8p–r). Hence, PDC activity is a key regulator of collagen production in CAFs and targeting PYCR1 effectively opposes this function.

## Discussion

Our work identified a link between proline biosynthesis and tumour ECM production in vivo, as we show that, in MCF10DCIS.com xenografts, tumours use circulating glutamine to generate proline for integration into collagen. Using different models of human mammary NFs and CAFs, we uncovered proline synthesis via PYCR1 as a major regulator of enhanced collagen production. Targeting PYCR1 in CAFs in cotransplantation models of breast cancer reduced tumour collagen and was sufficient to reduce tumour growth and metastasis. Therefore, we have identified PYCR1 as a candidate target to reduce tumour collagen to oppose breast cancer progression. It remains to be seen whether this is also the case when heterogeneous populations of CAFs populate the tumour stroma, and in other tumour types, such as PDAC, in which CAFs and collagen have been shown to have tumour-restraining functions^10,20,21^.

**Extended Data Fig. 8:**
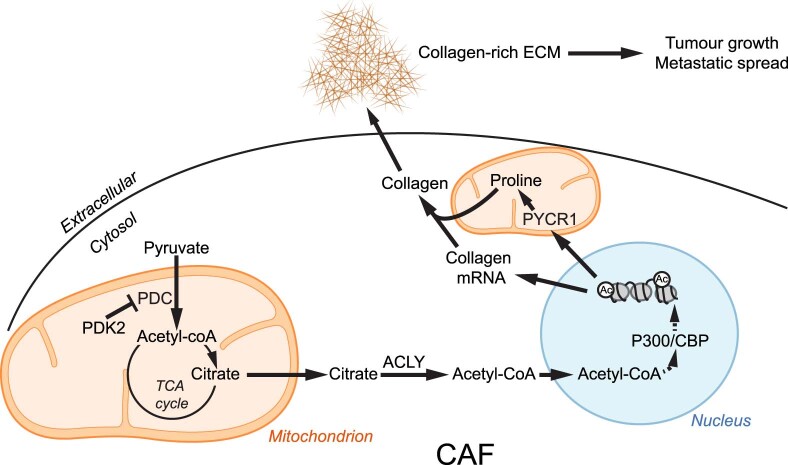
Working model. Scheme showing how PDH-derived acetyl-CoA and PYCR1-derived proline act together to stimulate and maintain collagen production in CAFs.

CAF metabolism influences tumour incidence and aggressiveness. Cancer cells can develop dependencies on amino acids produced and secreted by CAFs to fuel their growth^23–25,50^. For example, tumour collagen-derived proline promotes PDAC cell survival under nutrient-limited conditions^25^. Here we establish that proline is also important to support the CAF phenotype and provide evidence that enhanced proline synthesis through PYCR1 is a requirement for the biosynthesis of an abundant collagen-rich ECM (Extended Data Fig. 8). Our findings are consistent with the knowledge that collagens have an exceptionally high proline content^27^, and that fibroblasts in culture can generate proline from glutamine through PYCR1 (refs. ^51,52^). Interestingly, Schwörer and colleagues^52^ recently showed that targeting ALDH18A1, rather than PYCR1, reduced proline and collagen synthesis in TGFβ-stimulated fibroblasts because ALDH18A1 activity counteracts the mitochondrial redox potential generated by increased glucose and glutamine metabolism^52^. This suggests that, under acute TGFβ stimulation, fibroblasts increase collagen production to protect themselves from oxidative damage. In our NF and CAF models, however, we could not find consistent differences in glucose and glutamine usage, and targeting PYCR1 in CAFs resulted in reduced collagen in the stroma of tumour xenografts and in 2D and 3D cocultures with cancer cells. This suggests that increased collagen production in CAFs may not be a consequence of the redox imbalance described in acutely TGFβ-activated fibroblasts. We found no evidence from our metabolomics experiments that *PYCR1* expression affected redox balance in the CAFs. However, we cannot rule out the possibility that increased PDH activity in CAFs increases redox stress^53^ and PYCR1 upregulation counteracts this. Further experiments would be required to elucidate the connection between PYCR1 and redox balance in CAFs. However, as silencing PYCR1 affected ribosomal stalling in collagens, and we could rescue collagen translation and ECM production by providing exogenous proline, we propose that a major function of the proline synthesis pathway in CAFs is to provide proline residues for ECM production. Recently, Guo et al. found that PYCR1 levels in cancer cells may also play a role in ECM production in tumours; they showed that decreased levels of PYCR1 and proline correlated with significant reductions of tumour growth and collagen in the ECM^54,55^.

Importantly, we found that acetyl-CoA is another major metabolic regulator of collagen production in CAFs and a key epigenetic regulator. Increasing acetyl-CoA levels in fibroblasts induced H3K27 hyperacetylation and a transcriptional rewiring with elevated expression of pro-tumorigenic collagens, *COL1A1*, *COL6A1* and *PYCR1*, and EP300 is required for this process. Similarly to fibrosis, we also found that *COL1A1* and *COL6A1* genes are under the control of EP300 in CAFs^56^ and our chromatin immunoprecipitation analysis of EP300 further discovered that *PYCR1* expression may also be regulated by EP300. Recently, levels of pyruvate carboxylase-derived citrate have been shown to regulate H3K27 acetylation at enhancer and promoter regions of *Col1a1*, and collagen I expression in glutamine deprived TGFβ-activated fibroblasts^57^, further corroborating the link between citrate-derived acetyl-CoA and epigenetic control of collagen expression in fibroblasts.

We also found that *PDK2* is the most highly expressed of the four PDK isoenzymes in mammary NFs and a major regulator of PDC activity, acetyl-CoA production and gene expression rewiring in mammary fibroblasts. Since PDC-derived acetyl-CoA requires ACLY to contribute to the pool of nucleo-cytosolic acetyl-CoA, this implies that ACLY is an additional epigenetic regulator in CAFs. This is supported by our observation that pharmacological inhibition of ACLY reduced acetyl-CoA levels and H3K27 acetylation, correlating with reduced proline synthesis and production of collagen. Due to the challenge of performing metabolic tracing experiments in vivo, particularly in stromal cells that constitute a small proportion of the tumour, we have not validated in vivo whether, as in vitro, PDC activity is a major source of acetyl-CoA in CAFs. However, our analysis of gene expression in stroma dissected from normal and tumour tissue showed that *PDK2* expression is also reduced in samples from patients with primary breast cancer. Moreover, previous works reported decreased levels of PDK proteins in patient-derived non-small cell lung cancer stroma compared to normal lungs^58,59^, supporting the conclusion that PDC activity is enhanced in the stroma of malignant tumours. Future experiments are needed to confirm this hypothesis and to assess whether other metabolites contribute to the modulation of acetyl-CoA levels in CAF in vivo, such as acetate through ASSC2 or glutamine through reductive carboxylation.

In conclusion, our work uncovered that proline metabolism provides a critical link between the epigenetic regulation of collagen gene expression and protein production in CAFs, showing that CAF metabolism is a major vulnerability of pro-tumorigenic collagen production. This is an important finding, because *PYCR1* is among the top 20 metabolic genes overexpressed across cancer types, and proline synthesis has been proposed as a tumour-specific vulnerability^60,61^. Here, we show that *PYCR1* and collagen upregulation co-occur in many types of tumours, and anticipate that further work exploring PYCR1 as a therapeutic target to attack both cancer and stromal cells may result in further development of strategies to treat cancer. Collagen is the major contributor to the formation of desmoplastic tumour stroma. Thus, our study implies that targeting PYCR1 may also offer opportunities to tackle tumour-associated fibrosis to improve effective drug delivery and immune cell recruitment.

## Methods

### Ethical approval

Ethical approval for obtaining patient samples was given through the National Health Service (NHS) Greater Glasgow and Clyde Biorepository. All participants gave specific consent to use their tissue samples for research. All mouse procedures were in accordance with ethical approval from University of Glasgow or the Institutional Animal Care and Research Advisory Committee of the K.U. Leuven under the revised Animal (Scientific Procedures) Act 1986 and the EU Directive 2010/63/EU authorized through Home Office Approval (Project licence number 70/8645).

### Cell culture

The cancer cell-derived, immortalized human mammary CAFs and NFs (cCAFs and cNFs) were provided by A. Orimo. αSMA levels between paired NFs and CAFs was routinely monitored to ensure that during our experiments the NFs were not activated. Fibroblasts were cultured in DMEM supplemented with 10% foetal bovine serum (FBS), 2 mM glutamine and 1% penicillin/streptomycin. For physiol. DMEM experiments, fibroblasts were cultured in DMEM containing 5 mM glucose and supplemented with 0.65 mM glutamine, 100 µM pyruvate, 100 µM acetate, 10% FBS and 1% penicillin/streptomycin. MCF10DCIS.com cells were provided by P. Chavrier and cultured in F12 medium supplemented with 5% horse serum, 2 mM glutamine, 1% penicillin/streptomycin and 0.1% fungizone. Wood primary breast cancer cells were purchased from AMS Biotechnology Europe Ltd (AMSBIO) and cultured in Renaissance essential tumour medium (AMSBIO) supplemented with 5% FBS and 1% penicillin/streptomycin. For 2D and 3D cocultures, CAFs and cancer cells were mixed in a 1:1 ratio and cultured in a 1:1 mixture of DMEM and Renaissance essential tumour medium. For SILAC proteomics experiments, cCAFs and cNFs were cultured in SILAC DMEM supplemented with 2% FBS, 8% 10 kDa dialysed FBS, 2 mM glutamine and 1% penicillin/streptomycin. SILAC DMEM used for ‘light’ labelled cells contained 84 mg l^−1^ l-arginine and 146 mg l^−1^ l-lysine (Sigma), whereas the medium for ‘heavy’ labelled cells contained 84 mg l^−1^ ^13^C_6_^15^N_4_ l-arginine and 175 mg l^−1^ ^13^C_6_^15^N_2_ l-lysine. The following inhibitors were used to treat cells in culture: c646 (Sigma), A-485 (Tocris Bioscience), BMS303141 (Sigma) and CPI-613 (Sigma), PYCR1i^35^.

### pCAF and pNF isolation and immortalization

pCAFs and pNFs were isolated in house from patient samples. pCAF/NF2 were from an 88-year-old female patient with ER+, PR+, HER2− breast cancer and pCAF/NF3 were from a 79-year-old female patient with TNBC. From each patient, pCAFs were isolated from breast tumour tissue and pNFs from normal, tumour-adjacent tissue. Tissue was cut into small pieces, incubated in 10 mg collagenase A in DMEM overnight and fibroblasts isolated with a cell strainer. pCAFs and pNFs were immortalized using a human telomerase reverse transcriptase (hTERT)-expressing plasmid (pIRES2-hygro), provided by F. Calvo (IBBTEC, Santander). Lentivirus containing the hTERT plasmid was generated in HEK293T cells. Two rounds of viral transduction in fibroblasts were performed on consecutive days. Cells were selected using 50 μg/ml^−1^ hygromycin.

### Western blotting analysis

Cells were cultured for 48 h, following transfection and/or treatment with inhibitors and rescue compounds, then lysed in SDS buffer (2% SDS, 100 mM Tris-HCl pH 7.4). Proteins were separated using 4–12% gradient Bis-Tris gel (Life Technologies). Protein transfer was performed on methanol-activated polyvinylidene difluoride or nitrocellulose membrane. Western blot images were acquired using a myECL Imager (Thermo Scientific) and analysed using ImageStudioLite v.5.2.

### PDH activity assay

PDH activity was measured using the pyruvate dehydrogenase Enzyme Activity Microplate Assay Kit (Abcam catalogue no. ab109902) according to the manufacturer’s protocol.

### EdU proliferation assay

Cells were incubated in 1 µM EdU for 2 h and fixed in 4% PFA. EdU was fluorescently labelled using the Click-iT EdU Cell Proliferation Kit (Life Technologies) according to the manufacturers’ protocol, and nuclei were counterstained with 4,6-diamidino-2-phenylindole (DAPI). Images were acquired using a Zeiss 710 confocal microscope and ImageJ was used to count the number of total nuclei and EdU positive nuclei.

### Decellularized ECM preparation

Cells were seeded at 100% confluence on 0.2% gelatine, which was crosslinked using 1% glutaraldehyde, and then were cultured either for seven days with inhibitor treatment or three days if they had undergone transfection with siRNA or plasmids. ECM was decellularized with 20 mM NH_4_OH, 0.5% Triton X-100 in PBS. The ECM was washed in PBS with Ca^2+^ and Mg^2+^ and collected and lysed in SDS buffer (4% SDS, 0.1 M dithiothreitol (DTT), Tris-HCl pH 7.4).

### Cell transfection and infection

For transient expression or siRNA knockdown, 2 × 10^6^ fibroblasts were harvested and used in each transfection with a Nucleofector device (Lonza) according to the manufacturer’s protocol using the program T-20 and the Amaxa kit R (Lonza). Cells were transfected with 1–3 nM non-targeting siRNA as a control (GE Healthcare Dharmacon, catalogue no. D-001810-10-05) or with siRNAs targeting *PDK2* and *PYCR1* (Dharmacon, pool of four), with 5 µg pGCA-PDK2^N255A^ or pGCA-PDK2^WT^ (provided by A. McQuibban, University of Toronto) or with 5 µg pENTER-PYCR1 or pENTER plasmids (AMSBIO). Cells were used for experiments 48–72 h after transfection.

For stable knockdown of PYCR1 and PYCR2, shPYCR1 (Sigma catalogue no. TRCN000038983), shPYCR2 (Sigma catalogue no. TRCN0000046368) and shCTL (Sigma catalogue no. SHC016) lentivirus were generated in HEK293 cells. Two rounds of viral transduction in pCAF2 were performed on consecutive days. Cells were selected using 2 µg ml^−1^ puromycin.

### Reverse transcriptase polymerase chain reaction

For genomic DNA extraction from whole blood, red blood cells were lyzed in a hypotonic solution of 0.2% NaCl for 30 s and brought under isotonic conditions with 1.6% NaCl and 0.1% glucose. The remaining leucocytes and circulating tumour cells were washed once with PBS and genomic DNA was extracted from the pellet using the QIAmp DNA mini kit (Qiagen). Total RNA was isolated from cells after 48 h in culture, following transfection and/or with inhibitor and rescue compound treatment. RNA was isolated with the RNEasy mini kit (Qiagen) according to the manufacturer’s instructions. Complementary DNA was synthesized from 1 µg RNA. DNA was diluted to 10 ng µl^−1^ and 2 µl was used in each quantitative polymerase chain reaction with reverse transcriptase (RT–qPCR) reaction. Reactions were performed using a Quant Studio 3 PCR machine (Thermo Scientific).

### Chromatin immunoprecipitation

Immunoprecipitation reactions were carried out using the Cut&Run Assay Kit (Cell Signaling Technology). Then 2 × 10^5^ cells were used for each immunoprecipitation reaction. For ChIP–qPCR, input and immunoprecipitated DNA were quantified by real-time qPCR. Each ChIP DNA sample was normalized to the input DNA.

### MS-proteomic analysis

Cells were lysed in SDS buffer, or 100 µg tissue was homogenized in 4% SDS, 0.1 M DTT buffer. Proteins were precipitated with acetone and redissolved in 8 M urea. The proteins were then trypsin-digested. For SILAC experiments, equal quantities of heavy and light samples were mixed. Proteins were desalted by C18 StageTip^8^ before MS analysis.

For phosphorylated peptide enrichment, trypsin-digested peptides were acidified to pH 2.6 and acetonitrile (ACN) was added to a final concentration of 30%. The peptides were fractionated using an Akta system, using an increasing concentration of KCl in 5 mM KH_2_PO_4_ to 350 mM KCl. Each fraction was then enriched for phosphorylated peptides by incubation with TiO_2_ beads (GL Sciences) in the presence of 2,5-dihydroxybenzoic acid. Phosphorylated peptides were eluted with 15% ammonium hydroxide and 40% ACN, and desalted by C18 StageTip.

For acetylated peptide enrichment, cells were lysed in RIPA buffer (50 mM Tris-HCl pH 7.5, 150 mM NaCl, 1 mM EDTA, 1% NP-40, 0.1% sodium deoxycholate nicotinamide (10 mM) and trichostatin A (1 µM)). Proteins were precipitated with acetone and redissolved in 8 M urea. Equal quantities of heavy and light labelled proteins were combined and trypsin-digested. Acetylated peptides were enriched using with PTMScan Acetyl-Lysine Motif Kit (Cell Signaling Technology, catalogue no. 13416) according to the manufacturers’ protocol.

To analyse glutamine-derived proline incorporation into collagen, the cells were cultured in media containing 2 mM ^13^C_5_-glutamine for 72 h before ECM decellularization and collection. Each sample was separated on 4–12% gradient Bis-Tris gel (Life Technologies). The gel was sliced into three fractions, and each fraction was in-gel digested with trypsin.

Peptides were resuspended in 1% TFA, 0.2% formic acid buffer and injected on an EASY-nLC (Thermo Fisher Scientific) coupled online to a mass spectrometer. Peptides were eluted with a flow of 300 nl min^−1^ from 5 to 30% of buffer B (80% ACN, 0.1% formic acid) in a 60-min linear gradient. Eluted peptides were injected into an Orbitrap Elite, Q-Exactive HF or Orbitrap Fusion Lumos (Thermo Fisher Scientific) by electrospray ionization. MS data were acquired using XCalibur software (Thermo Fisher Scientific).

### MS-proteomic data analysis

The MS .raw files were processed with MaxQuant software and searched with the Andromeda search engine^8^. For SILAC experiments, multiplicity was set to 2, where the light labels were Arg0 and Lys0 and the heavy labels were Arg10 and Lys8. For the tracing experiments with ^13^C_5_-glutamine, ^13^C_5_-proline and ^12^C_5_-proline were added as heavy and light labels respectively. For label free quantification (LFQ) experiments, the LFQ setting was enabled. The false discovery rates at the protein and peptide levels were set to 1%. Specificity for trypsin cleavage was required and a maximum of two missed cleavages were allowed.

Perseus (v.1.5.0.36 for the phosphoproteome, v.1.5.5.1 for the acetylome and corresponding proteome and v.1.6.2.2 for total proteome) was used for downstream analysis. The data were filtered to remove potential contaminants, reverse peptides that match a decoy database and proteins only identified by site. To ensure unambiguous identification, only proteins identified with at least one unique peptide were considered.

### Estimation of kinase activities

KinAct^62^ is a computational method used to predict kinase activity scores from MS-based data. It infers an activity score for each protein kinase based on the regulation levels of phosphorylation events catalysed by this specific kinase. The method relies on prior knowledge of kinase/phosphatase-to-substrate relations and the kinase-substrate enrichment analysis method^63^ for the kinase activity estimation.

KinAct was applied to the phosphoproteomic SILAC-labelled NF and CAF data which was performed in two independent experiments. The log_2_ ratios of the two experiments were averaged and input into the KinAct pipeline.

### Metabolites extraction and LC–MS analysis

For tracing experiments, cells were labelled for 24 h with ^13^C_6_-glucose, ^13^C_5_-glutamine, ^13^C_3_-pyruvate or ^13^C_2_-acetate (100 µM). With the exception of acetate, which was supplemented to the media, the ^13^C-labelled metabolite replaced the concentration of the metabolite in DMEM. Intracellular metabolites were extracted with extraction buffer (aqueous solution of 50% methanol and 30% ACN). Blood samples were diluted 1:50 in extraction buffer and incubated with shaking at 4 °C. Tumour samples were homogenized at 4 °C in extraction buffer at a concentration of 20 mg ml^−1^ (tissue per extraction buffer). Samples were analysed using a Q-Exactive Orbitrap mass spectrometer (Thermo Scientific) in combination with a Thermo Ultimate 3000 HPLC system. Then 5 μl of cell extract was injected and the metabolites were separated over a 15 min mobile phase gradient from an initial ACN content of 80% ACN with 20% ammonium bicarbonate (pH 9.2) decreasing to 20% ACN with a flow rate of 200 μl min^−1^. The metabolites were detected over a period of 25 min using the Q-Exactive mass spectrometer across a mass range of 75–1,000 *m*/*z* and at a resolution of 35,000 (at 200 *m*/*z*). To detect acetyl-CoA, a single ion monitoring method was employed. The Q-Exactive mass spectrometer was used to monitor the three masses for acetyl-CoA labelled +0, +1 or +2 (810.1331, 811.1364 and 812.13976 *m*/*z*) with an isolation window of 0.7 *m*/*z* for each isotope. Peak identification and area quantification were carried out using TraceFinder software by comparison of the retention time and exact ion mass to that of authenticated standards.

### Ribosome profiling and analysis

30 × 10^6^ cells were treated with cycloheximide (100 μg ml^−1^) for 5 min and lysed in buffer A (20 mM Tris-HCl, pH 7.8, 100 mM KCl, 10 mM MgCl_2_, 1% Triton X-100, 2 mM DTT, 100 μg ml^−1^ cycloheximide, 1X complete protease inhibitor). Lysates were treated with 2 U μl^−1^ of RNase I (Ambion) for 45 min at room temperature. Lysates were fractionated on a linear sucrose gradient and the fractions enriched in monosomes were pooled. Ribosome protected fragments were purified using Trizol reagent (Invitrogen). Library preparation and differential ribosome codon reading (diricore) analysis were performed according to the method previously described^37^.

### Collagen quantification in monoculture and 2D cocultures

Cells were seeded at 100% confluence, either as a CAF monoculture or as a 1:1 coculture of CAFs and Wood primary breast cancer cells. The cells were cultured for 96 h in the presence of inhibitors or rescue compounds to allow accumulation of the matrix. Cells were incubated with 1 µM of the fluorescent collagen binding protein CNA35-mCherry^36^ for 1 h to label collagen, then fixed and counterstained with DAPI. Images were taken on a Zeiss 710 confocal microscope. Regions of CAFs were defined and collagen staining was quantified using ImageJ software.

### Microfluidic device design and preparation

Microfluidic devices were fabricated using previously established methods and used to culture spheroids^38^. Multilayer devices were composed of arrays of microfluidic channels, each of which was connected by two open wells. In short, the polydimethylsiloxane (PDMS) prepolymer (Sylgard 184, Dow Corning) and curing agent were combined in a 1:10 ratio and poured onto patterned silicon wafers. Once cured, the PDMS was removed from the wafers and open wells created using a 4 mm surgical biopsy punch (Miltex). Devices were cleaned and exposed to an oxygen plasma (Pico plasma cleaner, Diener electronic) to permanently bond the upper and lower PDMS layers together. Devices were incubated with a solution of 1% Synperonic F108 solution (Sigma Aldrich) to achieve ultra-low adhesion conditions.

### 3D coculture in microfluidic devices

Cells were seeded at a 1:1 ratio of Wood primary breast cancer cells: CAFs into devices at a concentration of 7 × 10^6^ cells per ml to form spheroids, with each microfluidic channel containing at least 32 spheroids of similar dimension (~80 µm diameter) for analysis. A cell suspension was injected in the open wells, which flowed into the microfluidic channels until they remained trapped into the microwells. Spheroids were formed within 24–48 h. Cells that were not trapped in microwells were removed from the device. Media with and without inhibitors and rescue agents was exchanged every 48 h for one week beginning 24 h after cell seeding.

### Collagen quantification in 3D cocultures

For visualization of total collagen, 1 µM CNA35-mCherry was incubated with the cells for a 2 h period. After this, cells were washed twice with PBS to ensure removal of any residual staining solution. PBS was then added again before imaging the devices.

An inverted microscope (Observer A1, Zeiss) connected to an Orca Flash 4.0 camera (Hamamatsu) was used to acquire bright field images of spheroids every 24–48 h. Epifluorescence microscopy was performed immediately after cell staining and image analysis carried out using ZEN Blue and Fiji.

### MCF10DCIS.com-CAF xenograft

Here 1.5 × 10^6^ pCAF2s expressing shCtl or shPYCR1 and 5 × 10^5^ MCF10DCIS.com in 200 µl 50% growth factor reduced phenol red free Matrigel in PBS were injected subcutaneously into the flank of 8-week-old female BALB/c nude mice (Charles River). Mice were randomly allocated to the two groups. For the ^13^C_5_-glutamine tracing experiment, 12 days after inoculation mice were injected intraperitoneally with 10 µl g^−1^ of 200 mM ^13^C_5_-glutamine and or with ^12^C_5_-glutamine. Mice were injected six times over 2 days, with the final injection 30 min before killing the mice. The mice were killed 14 days after inoculation and tumours were weighed and fixed in 4% PFA. The tumours were sliced into 400 µm sections and *Z*-stacks of each section were captured. The collagen was imaged using second harmonic generation microscopy in combination with confocal microscopy to detect GFP-expressing fibroblasts. Using ImageJ, regions of human CAFs were defined and the area of collagen surrounding the CAFs was quantified.

### Orthotopic 4T1 mammary tumour experiments

4T1 cells were transduced with a lentiviral vector encoding Akaluc, a firefly luciferase analogue with higher sensitivity, puromycin resistance and miRFP670. Transduced cells were sorted according to miRFP670 expression. Then 10^5^ 4T1-Akaluc cells and 10^6^ CAFs (shCtl or shPYCR1) were mixed in a volume of 50 μl of PBS and co-injected orthotopically to the right mammary fat pad of the second nipple of 6-week-old NMRI nu/nu female mice. To assess tumour hypoxia and tumour vessel perfusion, pimonidazole (60 mg kg^−1^, intraperitoneal) and fluorescein isothiocyanate-conjugated lectin (Lycopersicon esculentum; Vector Laboratories; 0.05 mg intravenous) were injected into tumour-bearing mice 1 h or 10 min before tumour harvesting, respectively.

### Histology analysis

Quantitative tissue analysis was performed on serial, formalin fixed paraffin embedded, mouse tumour sections using Halo software (v.3.1.1076.363, Indica Labs). For each of the two studies, software parameters were set which defined the stain of interest and all sections within each study were analysed using the same settings. Sirius Red, Pecam1 and Pimonidazole were given as a percentage area of the tumour with data normalized to the average of the control for each group.

### Gene expression analysis

The breast cancer (GSE90505) microarray datasets were downloaded from the Gene Expression Omnibus using the R statistical environment, v.3.5.0, and the Bioconductor package GEOquery, v.2.40.0 (ref. ^64^). Differential gene probe expression analysis was carried out using the linear models and differential expression for microarray data (Limma) package v.3.29.8 (ref. ^65^).

Gene expression data from Ma et al.^32^ were downloaded from Oncomine^66^. Probe g5902035_3p_a_at for *PYCR* and probe Hs.172928.0.A2_3p_a_at for *COL1A1* were used for the analysis.

### TCGA data analysis

TCGA data from the Pan Cancer Atlas study available in cBioportal were used for the analysis and were analysed with tools available in cBioportal. For each tumour type, the quartiles for the *PYCR1* and *COL1A1* genes were calculated based on the mRNA expression *Z*-scores relative to diploid samples. Tumours defined as *PYCR1* and *COL1A1* high were those that expressed the two genes at levels within their respective fourth (upper) quartile, while those define as *PYCR1* and *COL1A1* low were those that expressed the two genes at levels within the first (lower) quartile.

### scRNA sequencing

UMAPs were generated with Seurat v.4.0.2 and cell types annotated according to Wu et al.^3^.

### Calculation of amino acid content in ECM proteins

To calculate the amino acid composition of the human proteome, we retrieved all proteoforms from the Swiss-Prot section of UniProt database (release 2020_01). Lists of matrisome proteins were downloaded from the Matrisome Project^26^ and used to annotate the proteins. We developed a custom python script to calculate the amino acid frequencies in the entire proteome, the full matrisome and the individual core matrisome subcategories.

### Statistical analysis

GraphPad Prism v.6.0 was used for statistical analysis. Data were tested for normality, and for experiments with two conditions, a two-tailed unpaired *t*-test with Welch’s correction was used to determine the *P* value, while for experiments with more than two conditions, a one-way analysis of variance (ANOVA) test with Dunnett’s multiple comparison test was used. When data were not normally distributed, a Mann–Whitney test (two samples) and a Kruskal–Wallis with Dunn’s post hoc test (multiple comparison) were used instead. A *P* value of ≤0.05 was considered significant. All graphs show the mean ± s.e.m. of at least three biological replicates (independent experiments) unless otherwise stated. For MS-proteomic analysis, Perseus software was used for statistical analysis. A one-sample *t*-test for SILAC experiments or a two-sample *t*-test for LFQ experiments was used to determine significantly regulated proteins and enable data visualization as a volcano plot.

### Reporting summary

Further information on research design is available in the Nature Research Reporting Summary linked to this article.

## Supplementary information

Reporting Summary

Supplementary Table List of antibodies and primers used in the manuscript.

Supplementary Data 1 Amino acid count of the human proteome.

Supplementary Data 2 Ranking of proteins identified in the proteome of SDS soluble cCAF ECM.

Supplementary Data 3 Proteomic analysis of pCAF2-MCF10DCIS.com tumors and skin from 13C-glutamine infused mice.

Supplementary Data 4 COL1A1 peptides carrying 13C-labelled proline in cCAF siCtl and siPYCR1.

Supplementary Data 5 Acetylome and total proteome of mammary cCAF and cNF.

Supplementary Data 6 Proteome of cCAF treated with c646.

Supplementary Data 7 Phosphoproteome of mammary cCAF and cNF.

Supplementary Data 8 Prediction of activated and de-activated kinases in cCAF compared to cNF.

## Figures and Tables

**Extended Data Fig. 9:**
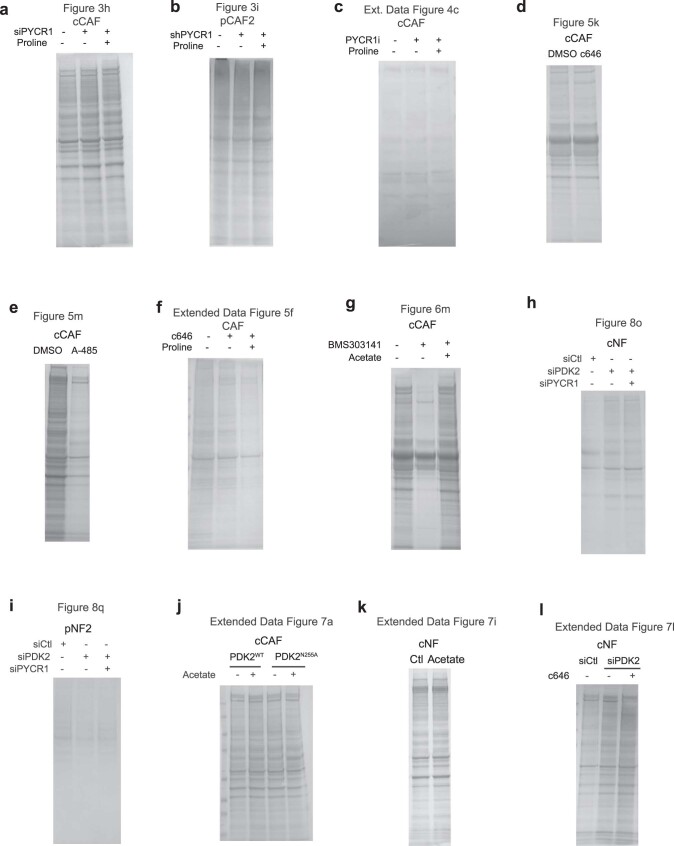
Loading controls for ECM western blots. **a**. Ponceau S total protein stain for blot shown in Fig. 3h. **b**. Ponceau S total protein stain for blot shown in Fig. 3i. **c**. Ponceau S total protein stain for blot shown in Extended Data Fig. 4c. **d**. Ponceau S total protein stain for blot shown in Fig. 5k. **e**. Ponceau S total protein stain for blot shown in Fig. 5m. **f**. Ponceau S total protein stain for blot shown in Extended Data Fig. 5f. **g**. Ponceau S total protein stain for blot shown in Fig. 6m. **h**. Ponceau S total protein stain for blot shown in Fig. 8o. **i**. Ponceau S total protein stain for blot shown in Fig. 8q. **j**. Ponceau S total protein stain for blot shown in Extended Data Fig. 7a. **k**. Ponceau S total protein stain for blot shown in Extended Data Fig. 7i. **l**. Ponceau S total protein stain for blot shown in Extended Data Fig. 7l.

## Data Availability

The .raw MS files and search/identification files obtained with MaxQuant have been deposited to the ProteomeXchange Consortium (http://proteomecentral.proteomexchange.org/cgi/GetDataset) via the PRIDE partner repository^67^ with dataset identifiers PXD018343 and PXD024746. All unique materials used are readily available from the authors. Publicly available datasets used in this study are Uniprot (https://www.uniprot.org/), the Matrisome Project (http://matrisomeproject.mit.edu/), METABRIC (http://www.cbioportal.org/study/summary?id=brca_metabric), TGCA (https://portal.gdc.cancer.gov/), Oncomine (https://www.oncomine.com/) and LCMD stroma (Gene expression omnibus accession code GSE90505). Source data are provided with this paper.

## Source data

Source Data Fig. 1 Statistical source data.
Source Data Fig. 2 Statistical source data.
Source Data Fig. 2 Unprocessed western blots.
Source Data Fig. 3 Statistical source data.
Source Data Fig. 3 Unprocessed western blots.
Source Data Fig. 4 Statistical source data.
Source Data Fig. 5 Statistical source data.
Source Data Fig. 5 Unprocessed western blots.
Source Data Fig. 6 Statistical source data.
Source Data Fig. 6 Unprocessed western blots.
Source Data Fig. 7 Statistical source data.
Source Data Fig. 7 Unprocessed western blots.
Source Data Fig. 8 Statistical source data.
Source Data Fig. 8 Unprocessed western blots.
Source Data Extended Data Fig. 1 Statistical source data.
Source Data Extended Data Fig. 1 Unprocessed western blots.
Source Data Extended Data Fig. 2 Statistical source data.
Source Data Extended Data Fig. 3 Statistical source data.
Source Data Extended Data Fig. 3 Unprocessed western blots.
Source Data Extended Data Fig. 4 Statistical source data.
Source Data Extended Data Fig. 4 Unprocessed western blots.
Source Data Extended Data Fig. 5 Statistical source data.
Source Data Extended Data Fig. 5 Unprocessed western blots.
Source Data Extended Data Fig. 6 Statistical source data.
Source Data Extended Data Fig. 6 Unprocessed western blots.
Source Data Extended Data Fig. 7 Statistical source data.
Source Data Extended Data Fig. 7 Unprocessed western blots.

## Extended data

Extended data is available for this paper at 10.1038/s42255-022-00582-0.

## Code availability

Codes for the estimation of kinase activities together with usage documentation are made available in http://saezlab.github.io/kinact/.
