# Author Correction: Cancer-associated fibroblasts require proline synthesis by PYCR1 for the deposition of pro-tumorigenic extracellular matrix

**DOI:** 10.1038/s42255-022-00632-7

**Published:** 2022-08-04

**Authors:** Emily J. Kay, Karla Paterson, Carla Riera-Domingo, David Sumpton, J. Henry M. Däbritz, Saverio Tardito, Claudia Boldrini, Juan R. Hernandez-Fernaud, Dimitris Athineos, Sandeep Dhayade, Ekaterina Stepanova, Enio Gjerga, Lisa J. Neilson, Sergio Lilla, Ann Hedley, Grigorios Koulouras, Grace McGregor, Craig Jamieson, Radia Marie Johnson, Morag Park, Kristina Kirschner, Crispin Miller, Jurre J. Kamphorst, Fabricio Loayza-Puch, Julio Saez-Rodriguez, Massimiliano Mazzone, Karen Blyth, Michele Zagnoni, Sara Zanivan

**Affiliations:** 1 Cancer Research UK Beatson Institute, Glasgow, UK; 2 Institute of Cancer Sciences, University of Glasgow, Glasgow, UK; 3 Centre for Microsystems and Photonics, EEE Department, University of Strathclyde, Glasgow, UK; 4 Laboratory of Tumor Inflammation and Angiogenesis, Center for Cancer Biology (CCB), VIB, Leuven, Belgium; 5 Laboratory of Tumor Inflammation and Angiogenesis, Department of Oncology, KU Leuven, Leuven, Belgium; 6 Translational Control and Metabolism, German Cancer Research Center (DKFZ), Heidelberg, Germany; 7 Heidelberg University, Faculty of Medicine, Institute for Computational Biomedicine, Bioquant, Heidelberg, Germany; 8 RWTH Aachen University, Faculty of Medicine, Joint Research Centre for Computational Biomedicine (JRC-COMBINE), Aachen, Germany; 9 Department of Pure and Applied Chemistry, Thomas Graham Building, University of Strathclyde, Glasgow, UK; 10 Rosalind and Morris Goodman Cancer Research Centre, McGill University, Montreal, Quebec Canada; 11 Department of Biochemistry, McGill University, Montreal, Quebec Canada; 12 Department of Medicine, McGill University, Montreal, Quebec Canada; 13 Department of Oncology, McGill University, Montreal, Quebec Canada

**Keywords:** Cancer microenvironment, Cancer metabolism, Metabolism

Correction to: *Nature Metabolism* 10.1038/s42255-022-00582-0, published online 27 June 2022

In the version of this article initially published, the surname of the third author, Carla Riera-Domingo, was misspelled as Riero-Domingo. The error has been corrected in the HTML and PDF versions of the article.

