# Supplementary material for: Cancer-associated fibroblasts require proline synthesis by PYCR1 for the deposition of pro-tumorigenic extracellular matrix
Source: Nat Metab. 2022 Jun 27;4(6):693–710. doi: 10.1038/s42255-022-00582-0 (PMC9236907; doi:10.1038/s42255-022-00582-0)
Supplement: Supplementary file 1 — Reporting Summary [file 42255_2022_582_MOESM1_ESM.pdf]

nature research

Corresponding author(s): Prof. Sara Zanivan

Last updated by author(s): May 5, 2022

Reporting Summary

Nature Research wishes to improve the reproducibility of the work that we publish. This form provides structure for consistency and transparency in reporting. For further information on Nature Research policies, see our Editorial Policies and the Editorial Policy Checklist.

Statistics

For all statistical analyses, confirm that the following items are present in the figure legend, table legend, main text, or Methods section.

n/a	Confirmed	
☐	☒	The exact sample size (*n*) for each experimental group/condition, given as a discrete number and unit of measurement
☐	☒	A statement on whether measurements were taken from distinct samples or whether the same sample was measured repeatedly
☐	☒	The statistical test(s) used AND whether they are one- or two-sided<br>*Only common tests should be described solely by name; describe more complex techniques in the Methods section.*
☒	☐	A description of all covariates tested
☐	☒	A description of any assumptions or corrections, such as tests of normality and adjustment for multiple comparisons
☐	☒	A full description of the statistical parameters including central tendency (e.g. means) or other basic estimates (e.g. regression coefficient) AND variation (e.g. standard deviation) or associated estimates of uncertainty (e.g. confidence intervals)
☐	☒	For null hypothesis testing, the test statistic (e.g. *F*, *t*, *r*) with confidence intervals, effect sizes, degrees of freedom and *P* value noted<br>*Give P values as exact values whenever suitable.*
☒	☐	For Bayesian analysis, information on the choice of priors and Markov chain Monte Carlo settings
☒	☐	For hierarchical and complex designs, identification of the appropriate level for tests and full reporting of outcomes
☐	☒	Estimates of effect sizes (e.g. Cohen's *d*, Pearson's *r*), indicating how they were calculated

Our web collection on statistics for biologists contains articles on many of the points above.

Software and code

Policy information about availability of computer code

Data collection	XCalibur 4.2 was used to collect mass spectra. Zen 3.0 software was used to acquire microscopy images.
Data analysis	GraphPad Prism 6 was used for statistical analysis. MaxQuant versions 1.6.6.3, 1.6.2.2, 1.5.5.1 and 1.3.8.2 and Perseus versions 1.6.2.2 and 1.6.0.7 were used to analyse proteomic data. TraceFinder 4.1 was used to analyse metabolomic data. The code used for kinase activity estimation can be found at http://saezlab.github.io/kinact/. ImageJ and Zen Blue 3.0 were used to analyse microscopy images. ImageStudio Lite 5.2 was used to analyse western blot images. Halo software v3.1.1076.363 was used to analyse IHC images. Gene expression data were analysed using R programming environment version 3.5.0 with RStudio, the Bioconductor package GEOquery, version 2.40.0 and Limma package version 3.29.8. TGCA were analysed in cBioportal.

For manuscripts utilizing custom algorithms or software that are central to the research but not yet described in published literature, software must be made available to editors and reviewers. We strongly encourage code deposition in a community repository (e.g. GitHub). See the Nature Research guidelines for submitting code & software for further information.

Data

Policy information about availability of data

All manuscripts must include a data availability statement. This statement should provide the following information, where applicable:

- Accession codes, unique identifiers, or web links for publicly available datasets
- A list of figures that have associated raw data
- A description of any restrictions on data availability

The .raw MS files and search/identification files obtained with MaxQuant have been deposited to the ProteomeXchange Consortium (http://proteomecentral.proteomexchange.org/cgi/GetDataset) via the PRIDE partner repository with dataset identifier PXD018343 and PXD024746. All unique materials used are readily available from the authors .raw MS data are associated to Figure 1b, Figure 1c, Figure 1f, Figure 1i, Figure 2a and Figure 5h. Publicly available

datasets used in this study are: Uniprot (https://www.uniprot.org/), the Matrisome Project (http://matrisomeproject.mit.edu/), METABRIC (http://www.cbioportal.org/study/summary?id=brca_metabric), TGCA (https://portal.gdc.cancer.gov/), Oncomine (https://www.oncomine.com/,), LCMD stroma (Gene expression omnibus accession code GSE90505), single cell RNA sequencing of TNBC (https://singlecell.broadinstitute.org/single_cell/study/SCP1106/stromal-cell-diversity-associated-with-immune-evasion-in-human-triple-negative-breast-cancer#study-download)

Field-specific reporting

Please select the one below that is the best fit for your research. If you are not sure, read the appropriate sections before making your selection.

[x] Life sciences   [ ] Behavioural & social sciences   [ ] Ecological, evolutionary & environmental sciences

For a reference copy of the document with all sections, see nature.com/documents/nr-reporting-summary-flat.pdf

Life sciences study design

All studies must disclose on these points even when the disclosure is negative.

Sample size	No statistical methods were used to predetermine sample size. We used a sample size of at least 3 for in vitro experiments and at least 6 mice or each in vivo experiment, which extensive experience has shown to be sufficient to determine reproducible results.
Data exclusions	No data were excluded from the analyses
Replication	Each experiment was replicated at least 3 times independently to confirm the results.
Randomization	For the in vivo experiments, mice were randomly allocated to each condition. For in vitro experiments, samples were randomly allocated to conditions.
Blinding	Blinding was not possible as the person who set up the experiment also carried out the analysis and data collection

Reporting for specific materials, systems and methods

We require information from authors about some types of materials, experimental systems and methods used in many studies. Here, indicate whether each material, system or method listed is relevant to your study. If you are not sure if a list item applies to your research, read the appropriate section before selecting a response.

Materials & experimental systems

n/a	Involved in the study	
[ ]	[x]	Antibodies
[ ]	[x]	Eukaryotic cell lines
[x]	[ ]	Palaeontology and archaeology
[ ]	[x]	Animals and other organisms
[ ]	[x]	Human research participants
[x]	[ ]	Clinical data
[x]	[ ]	Dual use research of concern

Methods

n/a	Involved in the study	
[x]	[ ]	ChIP-seq
[x]	[ ]	Flow cytometry
[x]	[ ]	MRI-based neuroimaging

Antibodies

Antibodies used	PDHA1 E1-alpha subunit, phospho S293 (abcam ab177461), PDHA1 E1-alpha subunit (abcam ab110334), PYCR1 (Proteintech 22150-1-AP), PYCR2 (Proteintech 17146-1-AP), ALDH18A1 (Proteintech 17719-1-AP), PYCR3 (Thermo Fisher TA502035) p300 (Cell signaling 54062), Histone H3, acetyl K27 (abcam ab4729), Histone H3 (Proteintech 17168-1-AP), Histone H3, acetyl 18 (Cell signaling 9675), Histone H3, acetyl K36 (abcam ab177179), Histone H2, acetyl K12 (abcam ab195494), Histone H2B (Proteintech 15857-1-AP), Histone H2, acetyl K5 (abcam ab45152), Collagen VI (abcam ab182744), PDK1 (abcam ab110025), PDK2 (Proteintech 15647-1-AP), PDK3 (abcam ab154549), PDK4 (abcam ab110336). Vinculin (Sigma V9131), GAPDH (Santa Cruz, sc-47724), β-tubulin (abcam ab179513), CD31 (abcam ab182981), HRP-conjugated secondary anti-mouse and anti-rabbit antibodies (NEB 7076, 7074)
Validation	All antibodies have been validated according to the manufacturer's website<br>PDHA1 E1-alpha subunit, phospho S293: Specificity for phosphorylated form was validated with phosphatase-treated membrane and human specificity validated with 293T cells https://www.abcam.com/pdha1-phospho-s293-antibody-epr12200-ab177461.html.<br>PDHA1 E1-alpha subunit: Specificty was validated with PDHA1 knockout and control HeLa cells. https://www.abcam.com/pdha1-antibody-9h9af5-ab110330.html<br>PYCR1: Specificity was validated with validated with HeLa cells with IgG as negative control. https://www.ptglab.com/products/PYCR1-Antibody-22150-1-AP.htm<br>PYCR2: Specificity was validated wtih HeLa and Jurkat cells and IgG as a negative control. https://www.ptglab.com/products/PYCR2-Antibody-17146-1-AP.htm<br>ALD18A1: Specificity was validated with HeLa and MCF7 whole cell lysate. https://www.ptglab.com/products/ALDH18A1-

Antibody-17719-1-AP.htm
PYCR3: Specificity was validated for western blot with whole cell lysates from 9 cell lines. https://www.thermofisher.com/antibody/product/PYCRL-Antibody-clone-OTI2E10-Monoclonal/TA502035
p300: Specificity was validated for western blot with HEK293 whole cell lysate and HCT-15 as a negative control. Use for ChIP was validated using SimpleChIP® Plus Enzymatic Chromatin IP Kit. https://www.cellsignal.co.uk/products/primary-antibodies/p300-d2x6n-rabbit-mab/54062
H3K27ac: Specificity for acetylated form was validated with Trichostatin A treated MEF cell lysate and human specificity validated with histone preparation from HeLa nuclear lysate. Use for ChIP was validated with chromatin from HeLa cells. https://www.abcam.com/histone-h3-acetyl-k27-antibody-chip-grade-ab4729.html
Histone H3: Specificity was validated with HEK293 cell lysate. https://www.ptglab.com/products/Histone-H3-Antibody-17168-1-AP.htm
H3K36ac: Specificity for acetylated form was validated with sodium butyrate treated HeLa whole cell lysate https://www.abcam.com/histone-h3-acetyl-k36-antibody-epr16992-ab177179.html
H3K18ac: Specificity was validated with NIH/3T3 cells, untreated or TSA-treated. https://www.cellsignal.co.uk/products/primary-antibodies/acetyl-histone-h3-lys18-antibody/9675?_=1651680110119&Ntt=9675&tahead=true
H2K12ac: Specificity was validated with HeLa whole cell and nuclear lysate, with unmodified recombinant histones as negative control. https://www.abcam.com/histone-h2b-acetyl-k12-antibody-chip-grade-ab195494.html
H2K5ac: Specificity for acetylated form was validated with mouse brain whole cell lysate. https://www.abcam.com/histone-h2a-acetyl-k5-antibody-ep856y-ab45152.html
Histone H2B: Specificity was validated with HEK293 cell lysate. https://www.ptglab.com/products/HIST2H2BE-Antibody-15857-1-AP.htm
Collagen VI: Specificity was validated with Collagen VI knockout and control HEK293 whole cell lysate human skeletal muscle whole cell lysate. https://www.abcam.com/collagen-vi-antibody-epr17072-ab182744.html
PDK1: Specificity was validated with jurkat and HeLa cell lysate. https://www.abcam.com/pdk1-antibody-4a11-ab110025.html
PDK2: Specificity was validated with human heart tissue and HeLa cells. https://www.ptglab.com/products/PDK2-Antibody-15647-1-AP.htm
PDK3: Specificity was validated with HepG2 cell lysate. https://www.abcam.com/pdk3-antibody-ab154549.html
PDK4: Specificity was validated with PDK4 knockout and control HeLa whole cell lysate and human heart tissue lysate. https://www.abcam.com/pdk4-antibody-1c2bg5-ab110336.html
Vinculin: Specificity was validated with HeLa and human fibroblast whole cell lysates. https://www.sigmaaldrich.com/GB/en/product/sigma/v9131?gclid=Cj0KCQjwyMiTBhDKARIsAAJ-9Vs6q7oMsNrS1D5Twbbm1xqwmUOkr4-WPc20KThUPu2E6wiKLz-spegaAs52EALw_wcB
GAPDH: Manufacturers' website states that human specificity was validated with HeLa, Hep G2, A549, U-87 MG and SK-BR-3 whole cell lysates. https://www.scbt.com/p/gapdh-antibody-0411?gclid=Cj0KCQjwyMiTBhDKARIsAAJ-9VuxX_t--d8vxwqkmz7DCDThgutpd0gwTS-V4bTtj9nLZ2t77x9UciwaAjeGEALw_wcB
β-tubulin: Specificity was validated with HeLa, Jurkat and A431 whole cell lysates. https://www.abcam.com/beta-tubulin-antibody-epr16774-ab179513.html
CD31: Specificity was validated with paraffin embedded mouse lung tissue https://www.abcam.com/cd31-antibody-epr17259-ab182981.html

Eukaryotic cell lines

Policy information about cell lines

Cell line source(s)	pCAFs/pNFs were derived from breast cancer patients. cCAFs/cNFs were kindly provided by Professor Akira Orimo (Juntendo University, Tokyo). Wood primary breast cancer cells were purchased from AmsBio. 4T1 and HEK 293T cells were purchased from ATCC. MCF10DCIS.com breast cancer cells were kindly provided by Professor Philippe Chavrier (Insitute Curie, Paris).
Authentication	None of the cell lines were authenticated
Mycoplasma contamination	All cell lines tested negative for mycoplasma
Commonly misidentified lines (See ICLAC register)	No commonly misidentified cell lines were used in this study

Animals and other organisms

Policy information about studies involving animals; ARRIVE guidelines recommended for reporting animal research

Laboratory animals	BALB/C nude mice, female, 8 weeks old or NMRI nude mice, female, 6 weeks old . Mice were housed in randomised groups of five, at 19°C to 23°C and 45 to 65% humidity with a 12-hour light-dark cycle, and were fed and watered ad libitum.
Wild animals	The study did not involve wild animals
Field-collected samples	The study did not involve field-collected samples
Ethics oversight	All mouse procedures were in accordance with ethical approval from University of Glasgow under the revised Animal (Scientific Procedures) Act 1986, the EU Directive 2010/63/EU authorised through Home Office Approval (Project licence number 70/8645) and the Institutional Animal Care and Research Advisory Committee of the K.U. Leuven.

Note that full information on the approval of the study protocol must also be provided in the manuscript.

Human research participants

Policy information about studies involving human research participants

Population characteristics	The human research participants were female. One patient (source of pCAF/NF2) was aged 88 and diagnosed with ER+ PR+ HER2- Grade 2 infiltrating ductal carcinoma, with lymph node infiltration. One patient (source of pCAF/NF3) was aged 75 and diagnosed with Triple negative Grade 3 invasive ductal carcinoma, with no lymph node infiltration.
Recruitment	Breast cancer patients undergoing mastectomy were recruited by the NHS Greater Glasgow and Clyde Biorepository. Both patients gave specific consent to use their tissue samples for research. We selected patients for which we had isolated fibroblasts from both matched tumour and normal adjacent breast tissue for fibroblast immortalisation, and further selected for invasive ductal carcinoma samples which could bias our results towards specificity for this breast cancer subtype.
Ethics oversight	The study protocol was in accordance with ethical approval from the NHS Greater Glasgow and Clyde Biorepository.

Note that full information on the approval of the study protocol must also be provided in the manuscript.
