# Supplementary figures and images for: Cancer-associated fibroblasts require proline synthesis by PYCR1 for the deposition of pro-tumorigenic extracellular matrix
Source: Nat Metab. 2022 Jun 27;4(6):693–710. doi: 10.1038/s42255-022-00582-0 (PMC9236907; doi:10.1038/s42255-022-00582-0)

Fig 2b

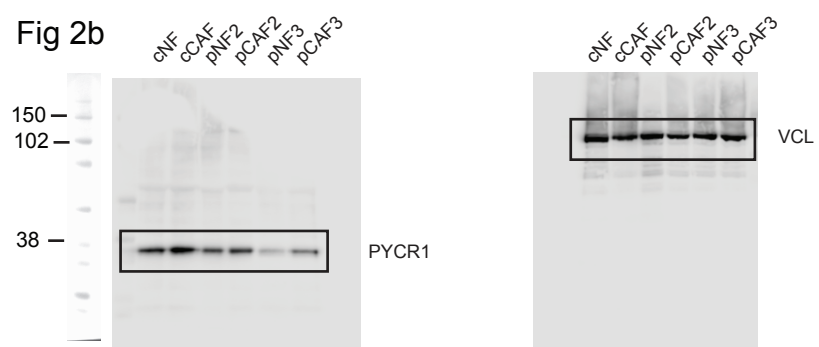

Supplement: Source Data Fig. 2 — Unprocessed western blots. [file 42255_2022_582_MOESM13_ESM.pdf]

Figure 3h

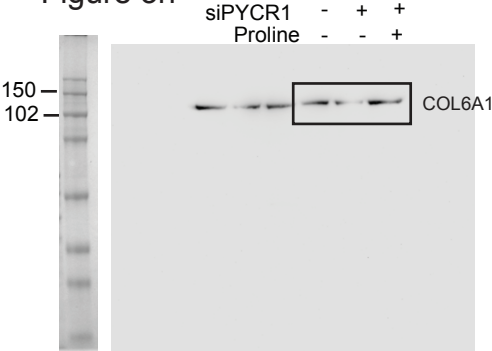

Figure 3i

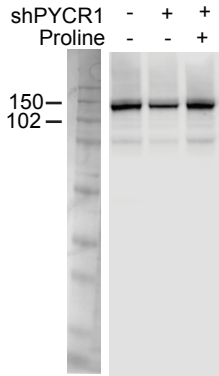

Supplement: Source Data Fig. 3 — Unprocessed western blots. [file 42255_2022_582_MOESM15_ESM.pdf]

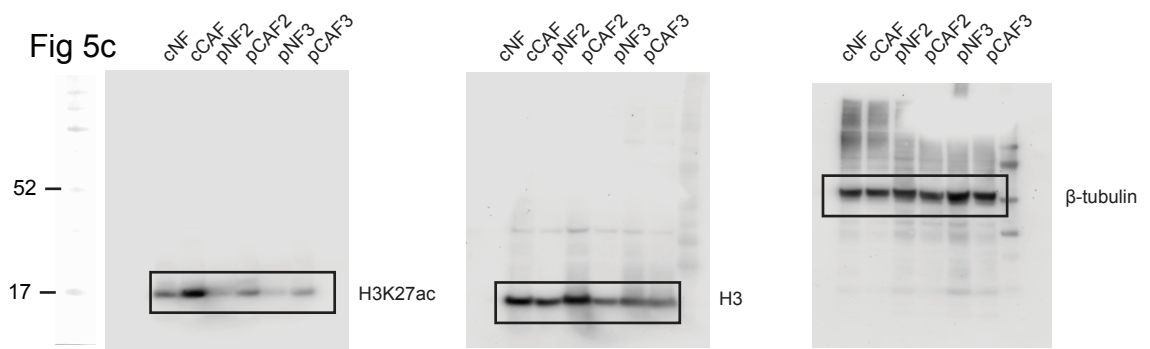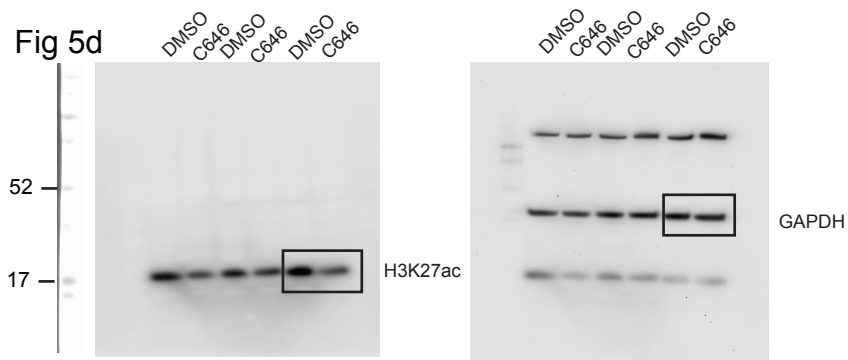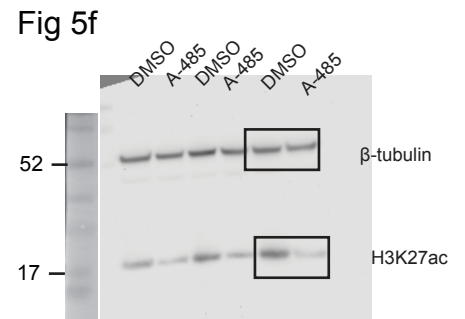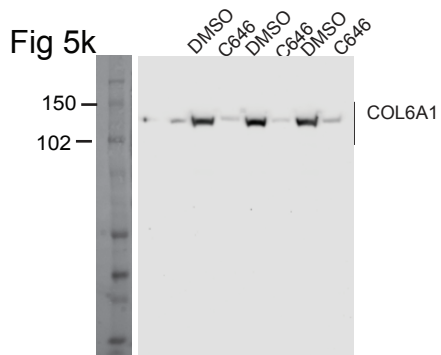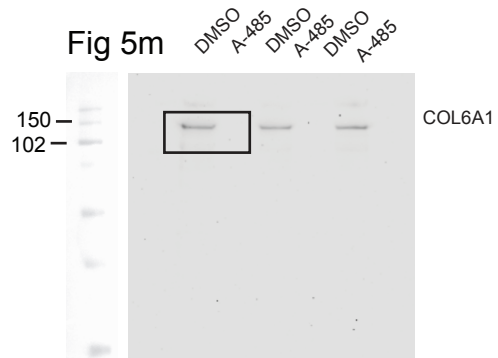

Supplement: Source Data Fig. 5 — Unprocessed western blots. [file 42255_2022_582_MOESM18_ESM.pdf]

Fig 6d

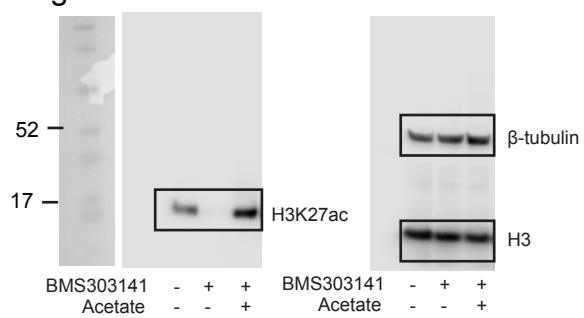

Fig 6f

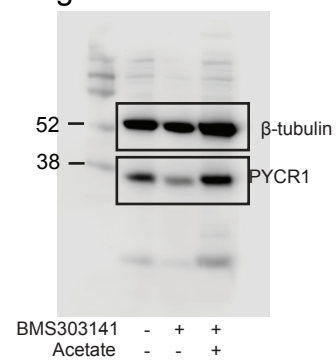

Fig 6m

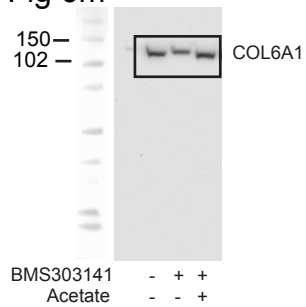

Supplement: Source Data Fig. 6 — Unprocessed western blots. [file 42255_2022_582_MOESM20_ESM.pdf]

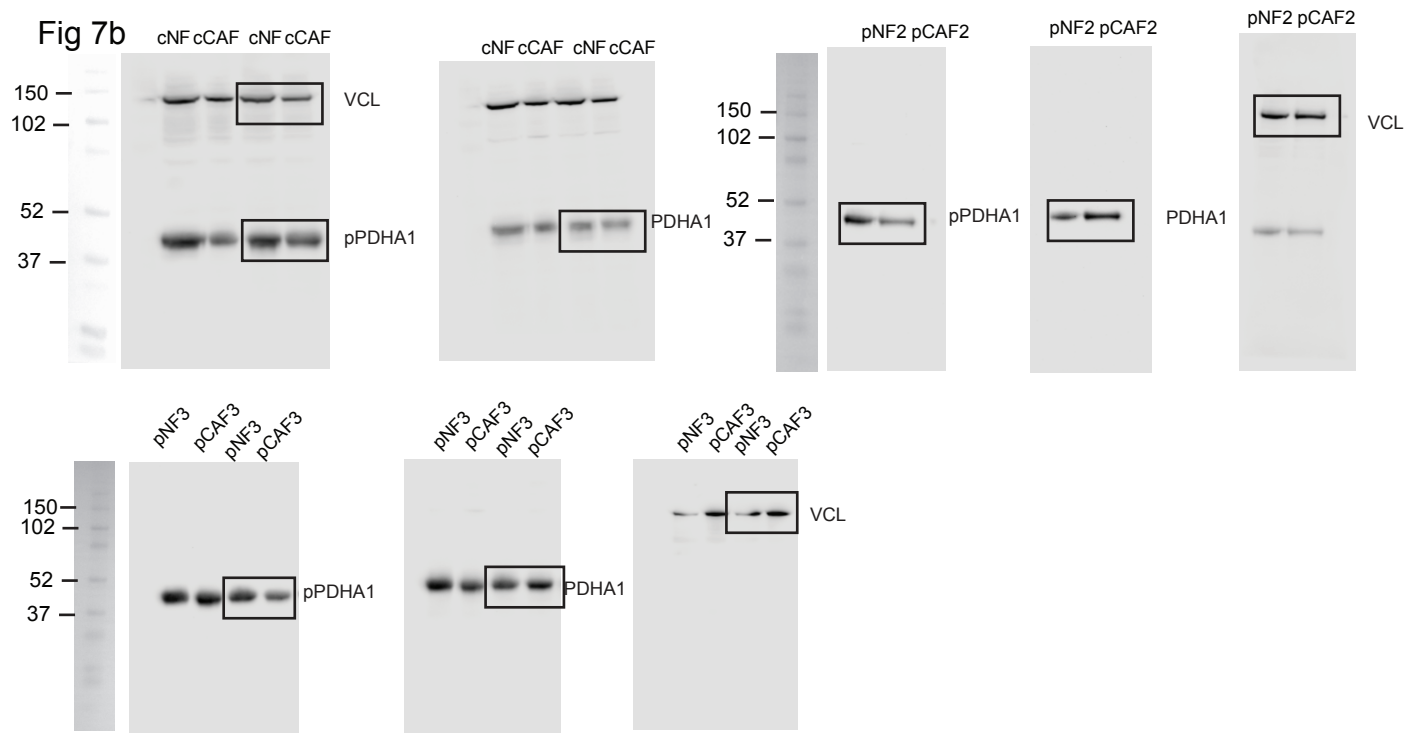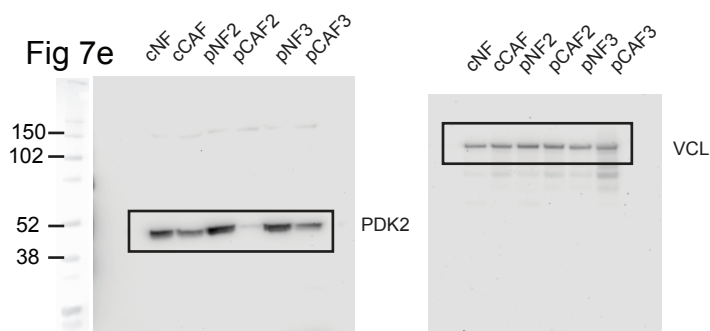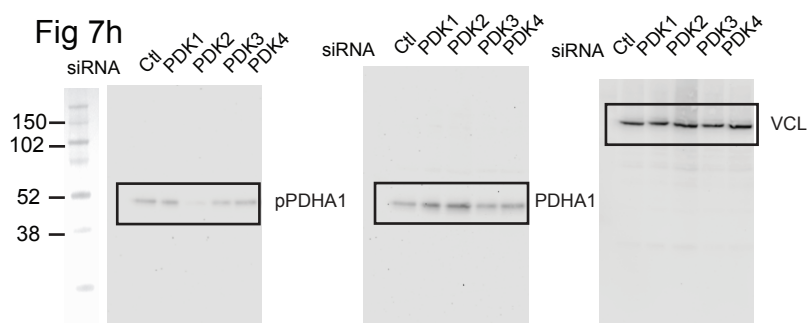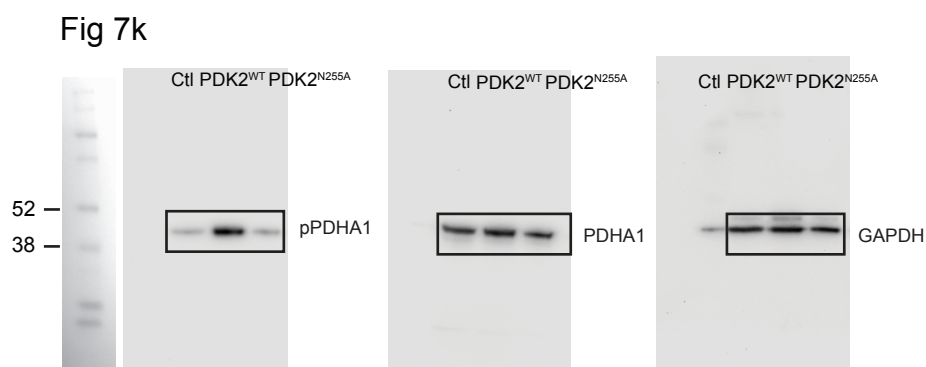

Supplement: Source Data Fig. 7 — Unprocessed western blots. [file 42255_2022_582_MOESM22_ESM.pdf]

Fig 8a

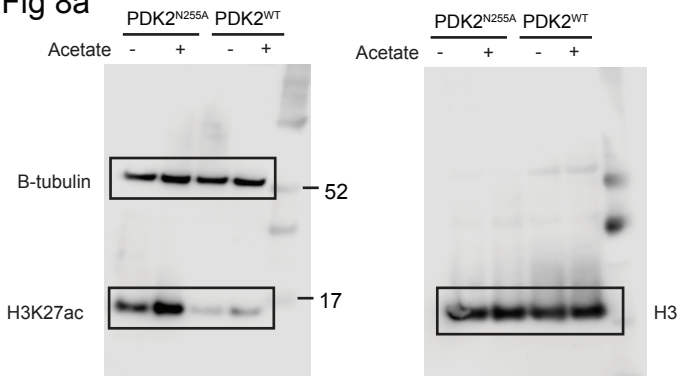

Fig 8c

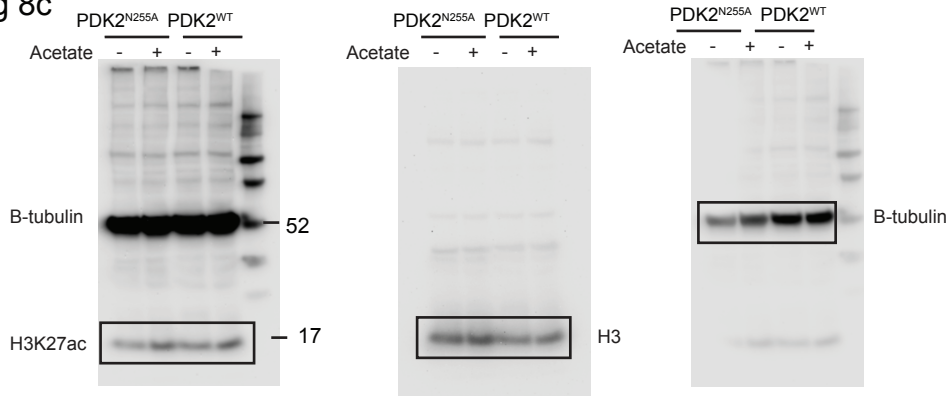

Fig 8h

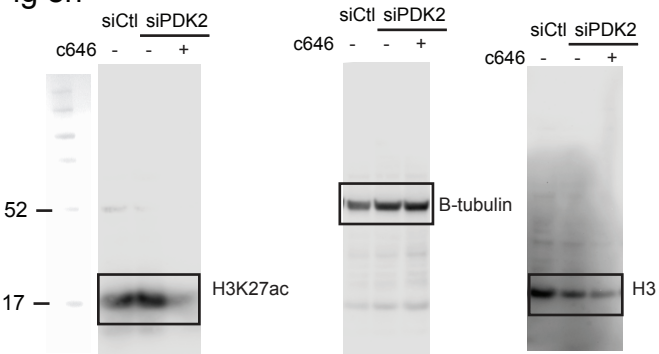

Fig 8j

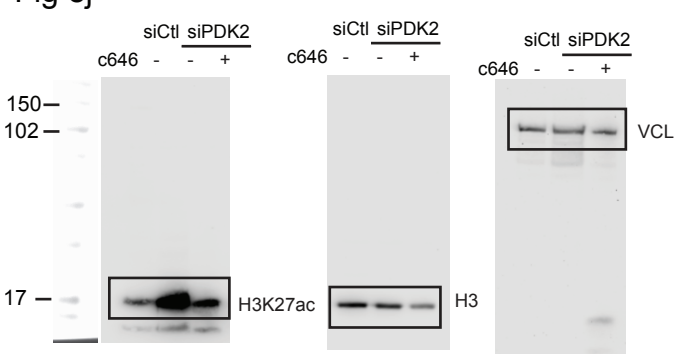

Fig 8o

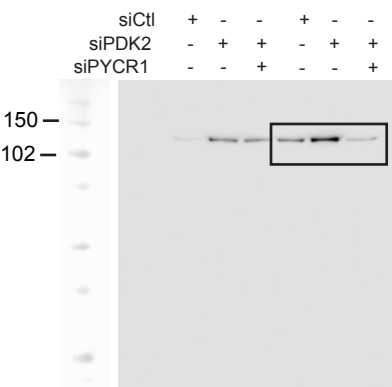

Fig 8q

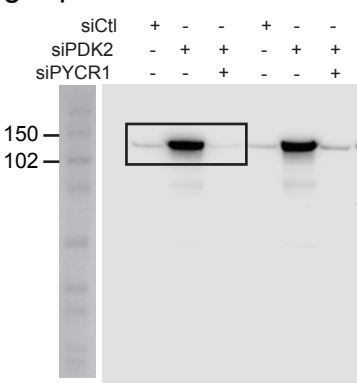

Supplement: Source Data Fig. 8 — Unprocessed western blots. [file 42255_2022_582_MOESM24_ESM.pdf]

Ext Fig 1c

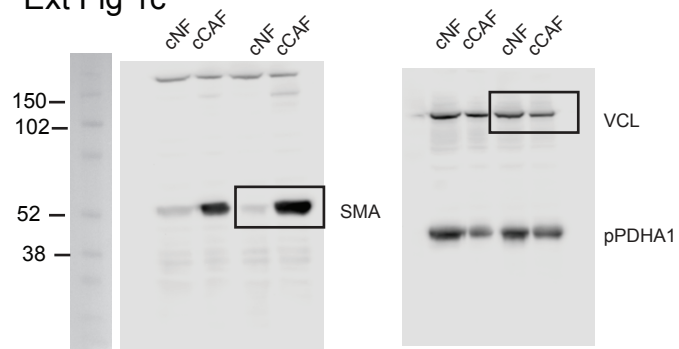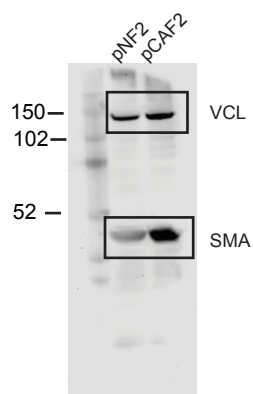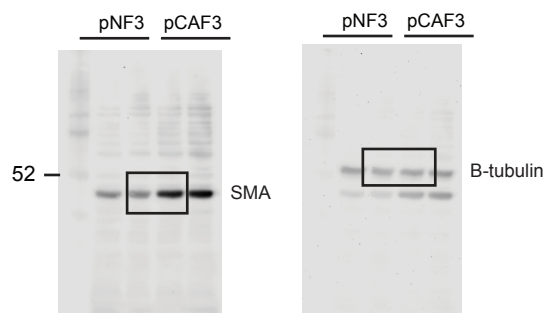

Supplement: Source Data Extended Data Fig. 1 — Unprocessed western blots. [file 42255_2022_582_MOESM26_ESM.pdf]

Ext Fig 3a

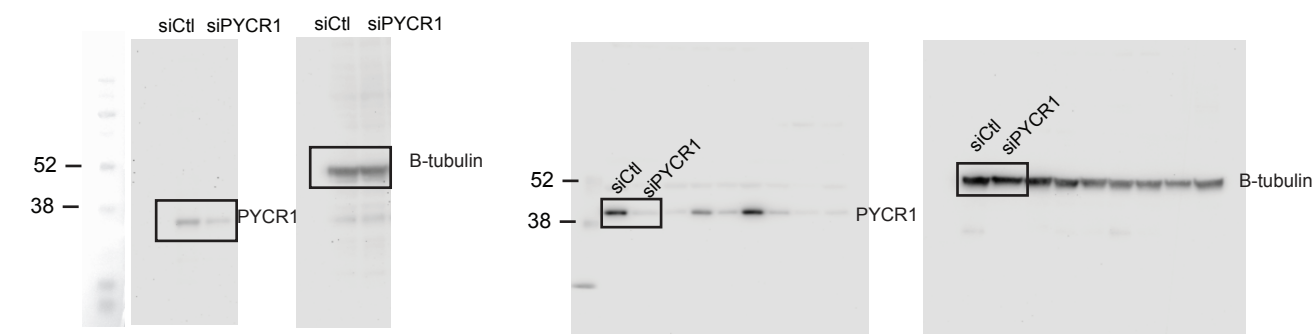

Ext Fig 3d

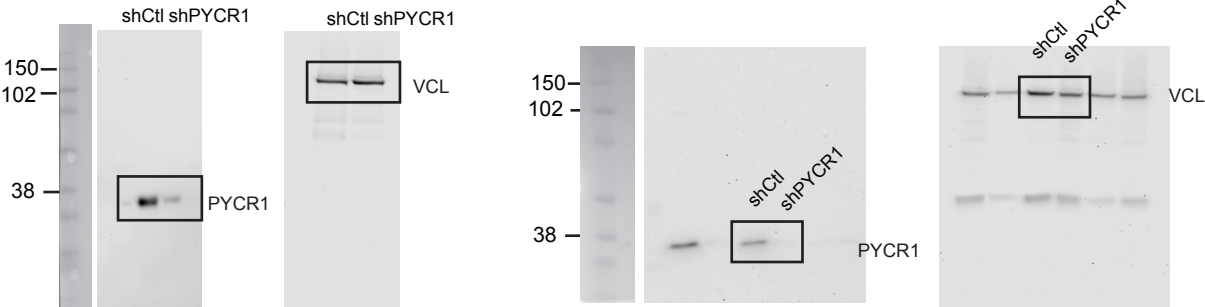

Ext Fig 3h

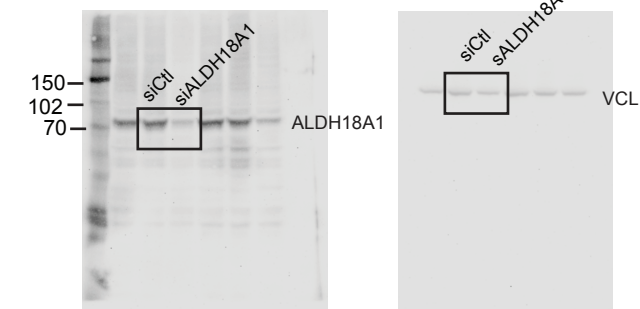

Ext Fig 3i

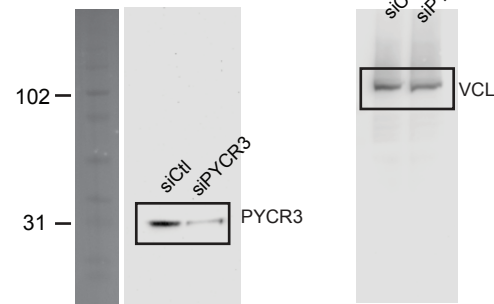

Ext Fig 3j

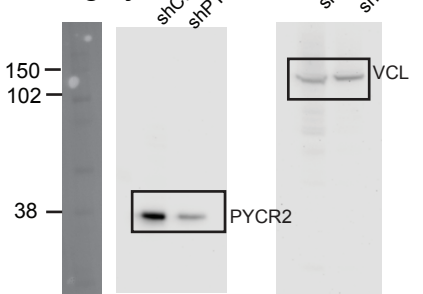

Ext Fig 3k

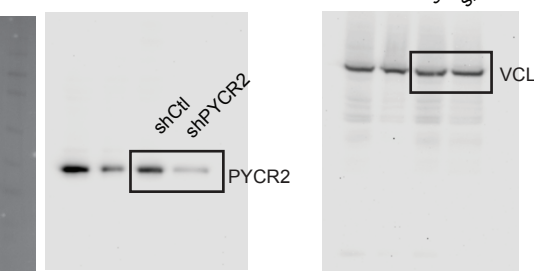

Ext Fig 3v

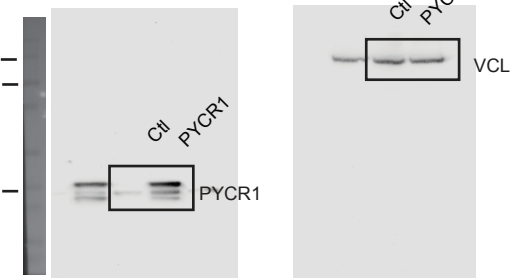

Supplement: Source Data Extended Data Fig. 3 — Unprocessed western blots. [file 42255_2022_582_MOESM29_ESM.pdf]

Ext Fig 4c

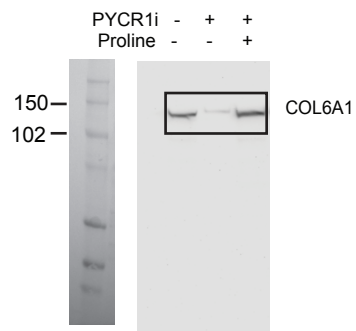

Ext Fig 4m

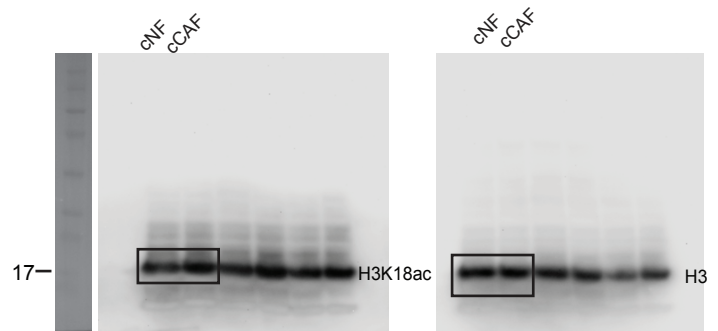

Ext Fig 4n

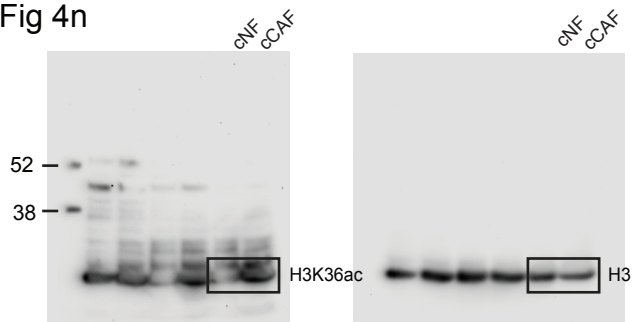

Ext Fig 4o

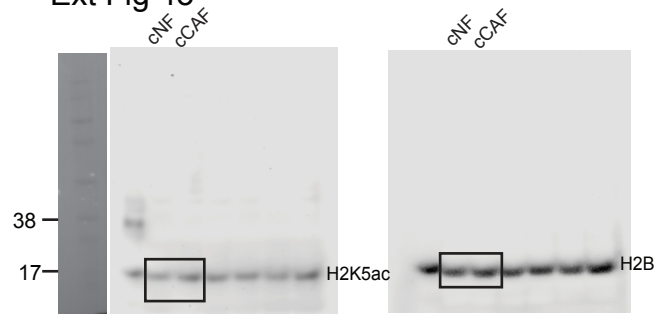

Ext Fig 4p

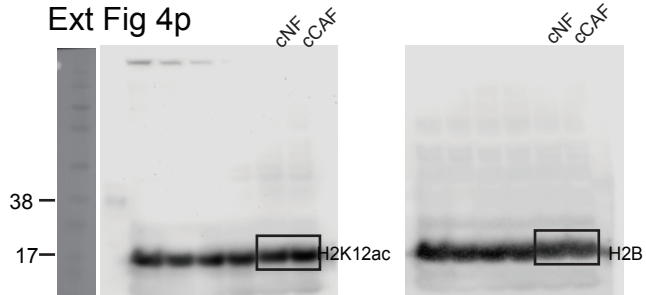

Ext Fig 4q

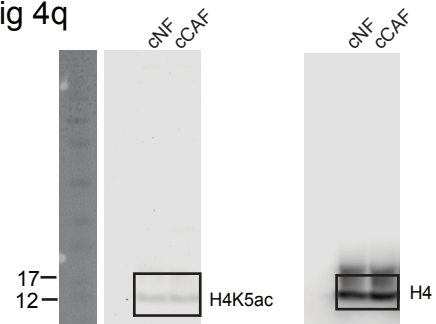

Supplement: Source Data Extended Data Fig. 4 — Unprocessed western blots. [file 42255_2022_582_MOESM31_ESM.pdf]

Extended Data Figure 5

Ext Figure 5f

Ext Figure 5e

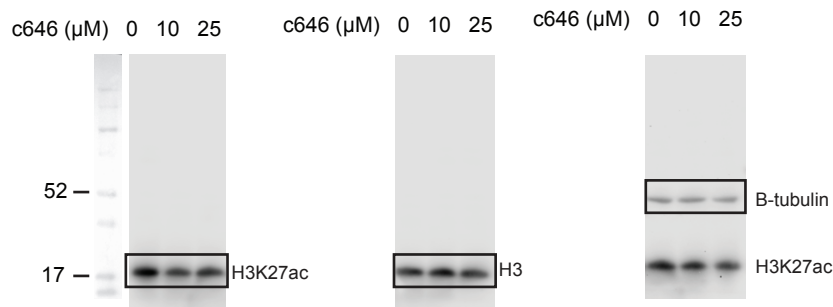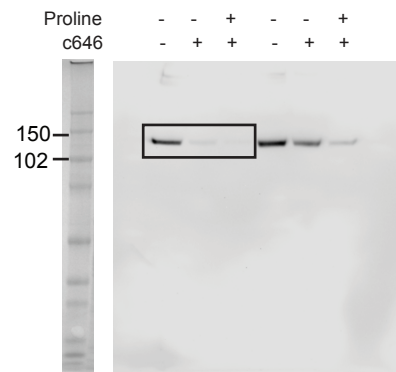

Ext Figure 5g

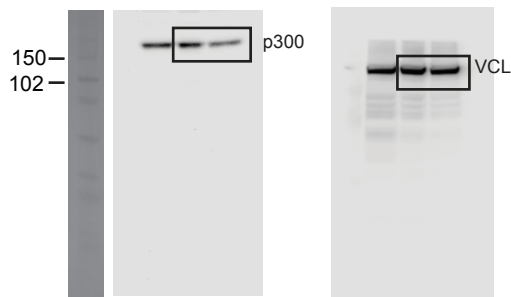

Ext Fig 5l

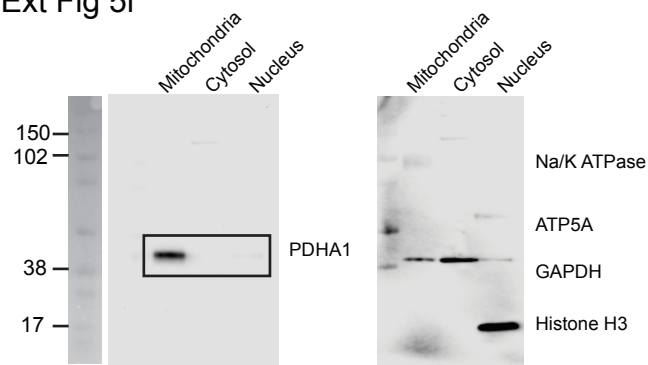

Ext Fig 5m

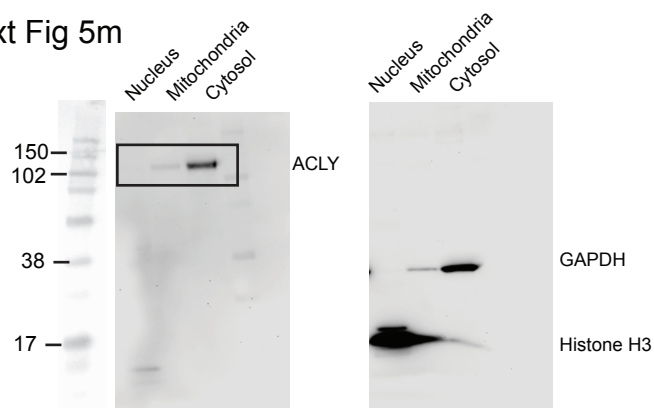

Ext Fig 5q

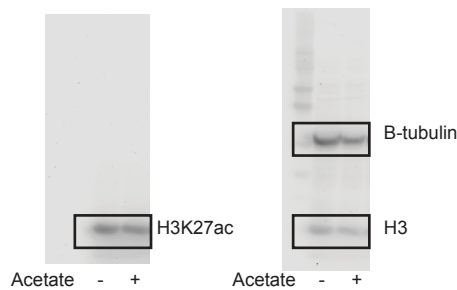

Supplement: Source Data Extended Data Fig. 5 — Unprocessed western blots. [file 42255_2022_582_MOESM33_ESM.pdf]

Extended Data Figure 6

Ext Fig 6a

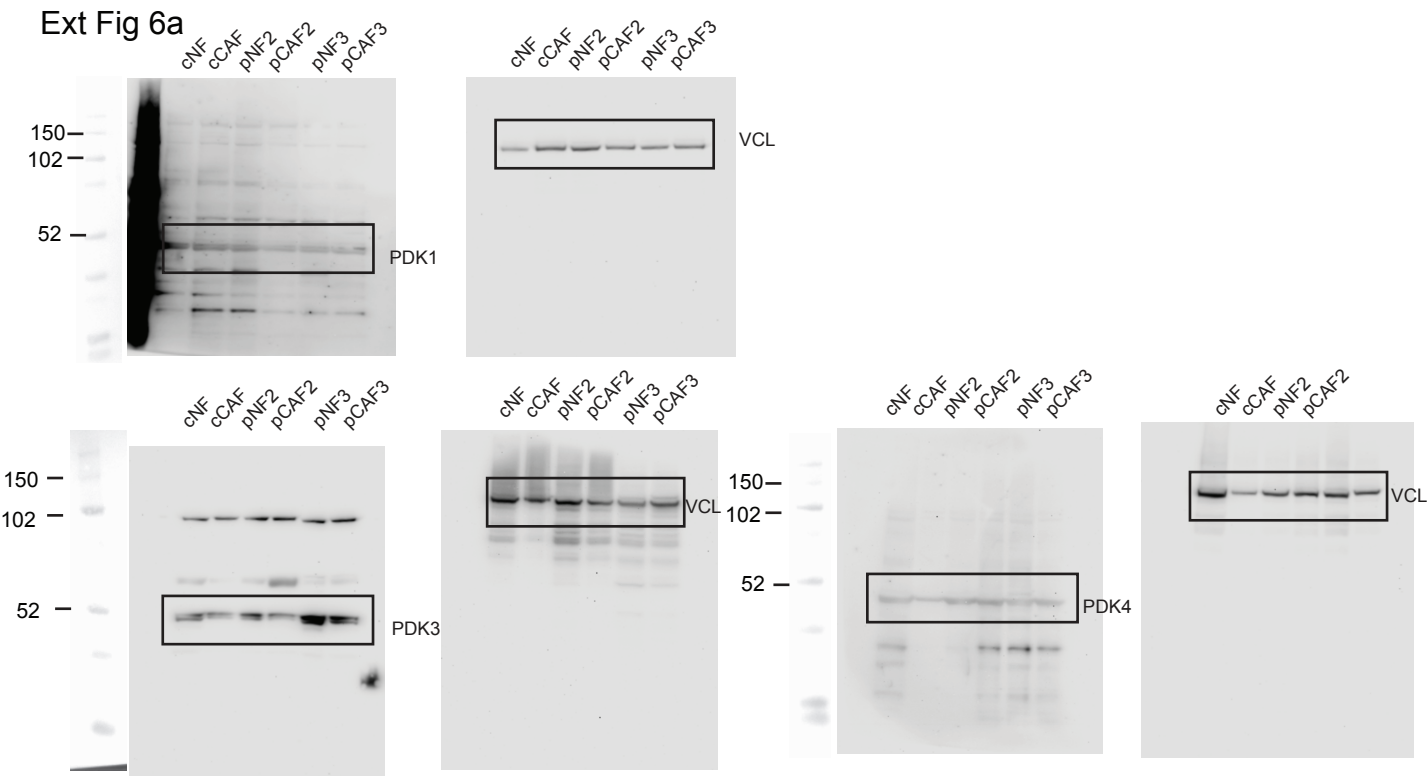

Ext Fig 6c

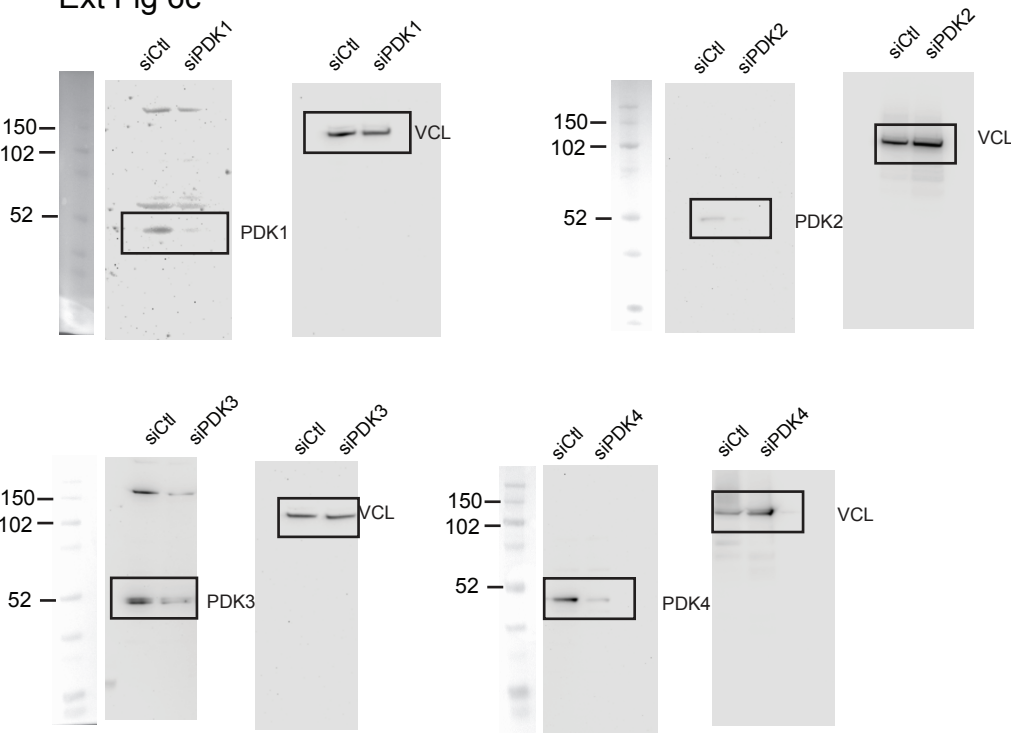

Ext Fig 6d

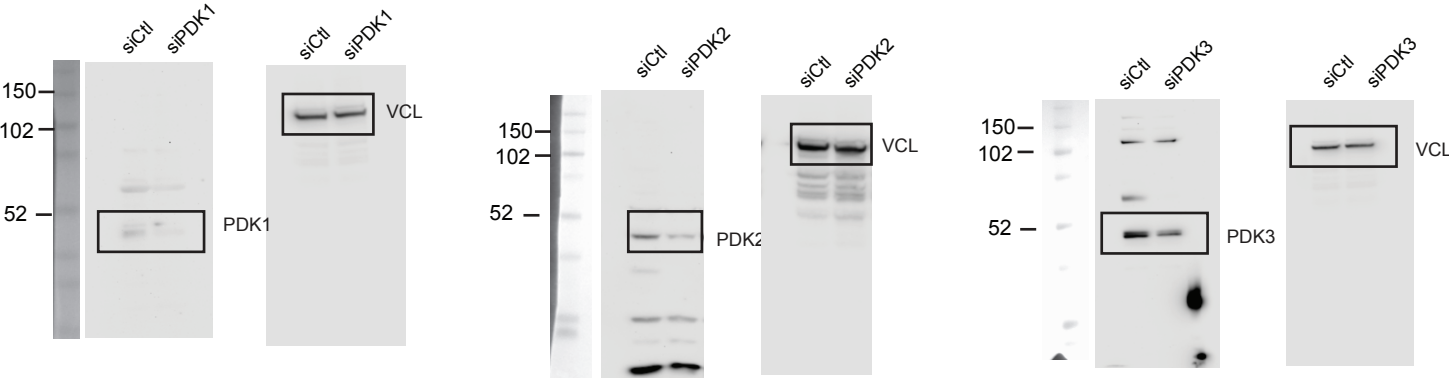

Supplement: Source Data Extended Data Fig. 6 — Unprocessed western blots. [file 42255_2022_582_MOESM35_ESM.pdf]

Extended Data Figure 7

Ext Fig 7a

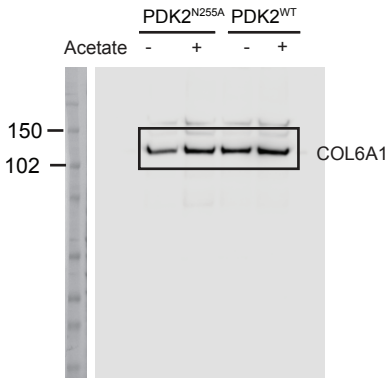

Ext Fig 7b

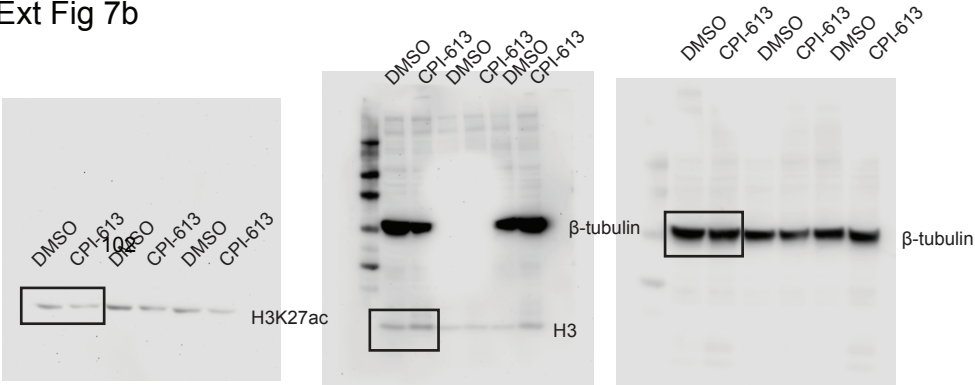

Ext Fig 7g

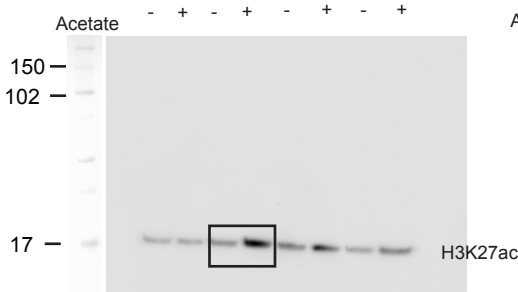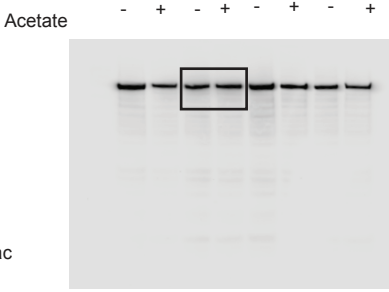

Ext Fig 7i

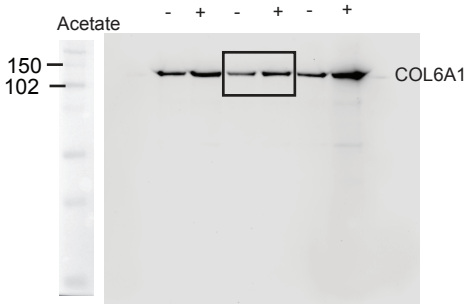

Ext Fig 7l

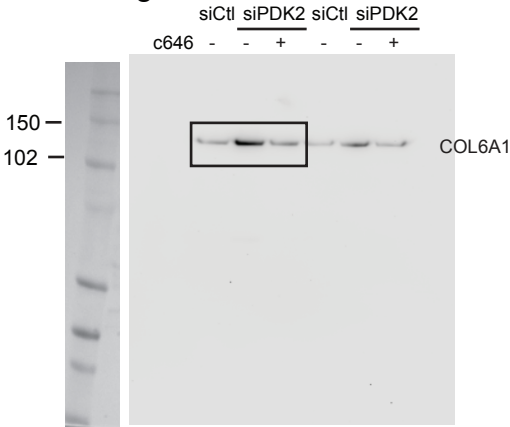

Ext Fig 7m

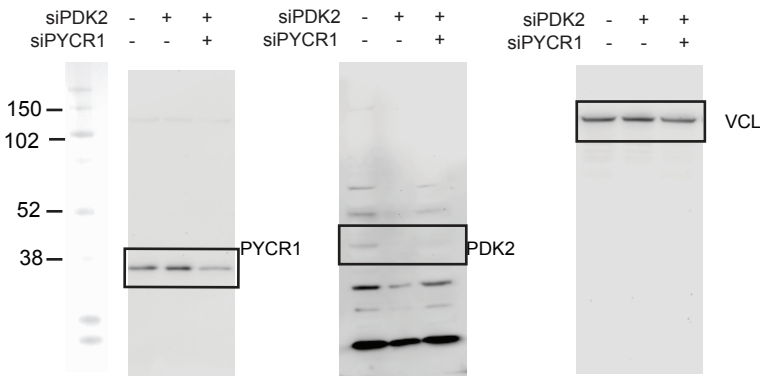

Ext Fig 7n

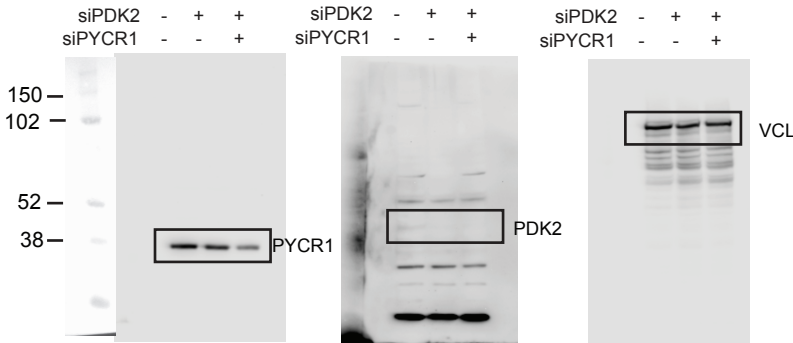

Supplement: Source Data Extended Data Fig. 7 — Unprocessed western blots. [file 42255_2022_582_MOESM37_ESM.pdf]
